# Cohort profile of the Heidelberg study on diabetes and complications HEIST-DiC

**DOI:** 10.1038/s41598-025-15343-8

**Published:** 2025-08-12

**Authors:** Elisabeth Kliemank, Ekaterina von Rauchhaupt, Lukas Seebauer, Mani Roshan, Malin Ansmann, Viktoria Flegka, Lukas Schimpfle, Dimitrios Tsilingiris, Hannelore Bartl, Thomas Fleming, Zoltan Kender, Johann M. E. Jende, Christoph M. Mooshage, Daniel Schwarz, Martin Bendszus, Peter Schirmacher, Stephan Herzig, Peter P. Nawroth, Stefan Kopf, Julia Szendroedi, Alba Sulaj

**Affiliations:** 1 Department of Endocrinology, Diabetology, Metabolic Diseases and Clinical Chemistry (Internal Medicine I), Heidelberg University Hospital, Heidelberg, Germany; 2 German Center for Diabetes Research (DZD), München-Neuherberg, Germany; 3 Department of Neuroradiology, Heidelberg University Hospital, Heidelberg, Germany; 4 Institute of Pathology, Heidelberg University Hospital, Heidelberg, Germany; 5 Institute for Diabetes and Cancer, Helmholtz Center Munich, Neuherberg, Germany; 6 Joint Heidelberg-IDC Translational Diabetes Program, Internal Medicine I, Heidelberg University Hospital, Heidelberg, Germany; 7 Chair Molecular Metabolic Control, Technical University Munich, Munich, Germany; 8 Joint Heidelberg-IDC Translational Diabetes Program, Helmholtz Center Munich, Neuherberg, Germany

**Keywords:** Diabetes-associated complications, Deep metabolic and clinical phenotyping, Identification of diabetes subtypes, Diabetes, Pre-diabetes, Diabetes, Pre-diabetes

## Abstract

The Heidelberg Study on Diabetes and Complications (HEIST-DiC) is a prospective longitudinal study focused on the development and progression of diabetes-associated complications. Participants with/without diabetes mellitus undergo annual phenotyping of diabetes-associated complications over 11 years. Assessments include: albuminuria, estimated glomerular filtration rate for chronic kidney disease; clinical neuropathy scores, Purdue Pegboard test, electrophysiological examination, transcutaneous electrical nerve fiber stimulation, quantitative sensory testing and high-resolution magnetic resonance neurography for distal sensorimotor polyneuropathy; heart rate variability for cardiovascular autonomic neuropathy; funduscopic examination of undilated pupils for retinopathy; the 6-minute walk test, spirometry, body plethysmography, and carbon monoxide-based diffusing capacity measurements for respiratory lung disease; non-invasive scores, transient elastography and hepatic ultrasound for metabolic dysfunction-associated steatotic liver disease; ankle-brachial index and carotid intima-media thickness for peripheral atherosclerosis; hand grip strength for muscle function; bioelectrical impedance analysis for body composition; skin autofluorescence for measurement of advanced glycation end products. Beta-cell function and tissue-specific insulin sensitivity are evaluated using oral glucose tolerance test or euglycemic hyperinsulinemic clamp. The biobank stores specimens of blood, urine, skeletal muscle, subcutaneous adipose tissue, and skin. Health-related quality of life, physical health, and somatic and depression symptoms are measured via standardized questionnaires. HEIST-DiC explores diabetes onset in high-risk individuals, disease progression and the development of complications, aiming to design personalized strategies to prevent, mitigate, or reverse diabetes-related complications.

**Trial registration:** The study was retrospectively registered at Clinicaltrials.gov (NCT03022721, date of registration 20170112).

**Supplementary Information:**

The online version contains supplementary material available at 10.1038/s41598-025-15343-8.

## Introduction

### Why was the cohort set up?

Diabetes mellitus (DM) remains a leading cause of blindness, kidney failure, lower limb amputation, heart attack, and stroke. Its global prevalence has surged, with over half a billion people currently affected^1,2^. Despite advances in DM treatment, particularly with the use of sodium-glucose cotransporter-2 (SGLT-2) inhibitors and glucagon-like peptide 1 (GLP-1) agonists for type 2 diabetes (T2D), glucose-centered therapies moderately reduce cardiovascular risk while at the same time increasing the risk for hypoglycemia^3^. The aim of this cohort is to identify DM subtypes with distinct courses of disease related to complications. Additionally, this cohort seeks to investigate new associations between DM subtypes and diseases previously not attributed to DM. This will aid clinical decision-making and enable the development of personalized treatments to prevent, or potentially reverse diabetes-associated complications.

### Complications of DM

The traditional classification of chronic diabetes-associated complications is based on the consequences of vascular damage and divides them into two main groups: microvascular and macrovascular complications^4^. While this classification is convenient in clinical practice, emerging evidence suggests it is limited, as diabetes-related damage extends beyond the vascular system, impacting nearly all organs, tissues and cells^5^. Microvascular complications are not solely due to microvascular changes, but other changes beyond the vascular bed, such as retinal neurodegeneration in retinopathy^6^, tubulointerstitial changes in nephropathy^7^ and neuronal deficits in neuropathy^8^, also play key roles. Additionally, impaired angiogenesis of large artery microvessels, a form of microangiopathy, contributes to the development of the diabetic macroangiopathic disease^9–11^. There is also increasing recognition of nonclassical diabetes-associated complications, including abnormal pulmonary function, metabolic dysfunction-associated steatotic liver disease (MASLD), cardiomyopathy, increased risk of carcinogenesis^5^, increased diabetes-related distress and impairment of psychological stress response^12,13^. By using a holistic approach, the HEIST-DiC cohort has the potential to investigate both classical and nonclassical diabetes-associated complications.

### Diabetes subtypes and risk of complications

Efforts to better understand the heterogeneity of DM have led to a paradigm shift, classifying individuals with newly diagnosed adult-onset DM into five clusters based on six simple clinical variables^14,15^. This new classification is significant as it stratifies individuals with recent-onset DM (diagnosis of DM within the past 12 months) into subgroups with varying risks for diabetes-associated complications. Particularly, individuals with severe insulin-resistant diabetes (SIRD) are at higher risk for cardiovascular, kidney and fatty liver disease, while those with severe insulin-deficient diabetes (SIDD) are more prone to diabetic neuropathy, often already present at disease onset^14,15^. Another retrospective cohort study found an increased risk of diabetic retinopathy (DR) in the SIDD cluster and a higher risk of nephropathy in the SIRD cluster^16^. The concept of new diabetes classifications provides deeper insights into disease heterogeneity but raises several unresolved questions, including: (i) the statistical methods used to define clusters, (ii) the stability of clusters over time, (iii) the use of genotypic versus phenotypic datasets, (iv) generalizability across different ethnic groups, (v) clinical relevance of in some cases only marginal absolute differences between subtypes and (vi) applications in clinical care^17^.

Early studies used k-means clustering at diabetes onset, assigning individuals to fixed clusters^14,15,18,19^. This approach broadly validated novel diabetes subgroups across Chinese, US, and non-white Emirati populations, suggesting potential generalizability across ethnicities^20,21^. However, rigid clusters at diagnosis fail to account for dynamic, longitudinal changes driven by initial pathological mechanisms. A German study found that 23% of individuals switched clusters within five years after diabetes onset, raising concerns about the practical utility of this rigid partitioning in managing ongoing diabetes^15^.

A novel soft-clustering method for patient stratification in ongoing diabetes identified a subgroup with obesity, insulin resistance, dyslipidemia, impaired β-cell sensitivity, rapid disease progression, and higher need for anti-diabetic therapy^22^. Another soft-clustering approach using genotypic data identified five robust clusters of T2D pointing to disease mechanisms reflected by clinical traits^23^. Advanced methods, such as a tree-like graph structure using reversed graph-embedded dimensionality reduction, enable stratification of pathophysiological components and diabetes-related complications throughout the course of diabetes^24^.

A clustering approach in individuals with prediabetes (PRED), who have an increased risk for developing T2D, identified six subtypes based on pathophysiological parameters. Of these, only two had a high imminent risk for developing T2D, despite impaired glucose metabolism^25^. Notably, one subtype exhibited an increased risk for kidney disease and all-cause mortality, despite only moderate risk for T2D^25^. Furthermore, diabetes-associated complications are already present in some individuals with PRED. Similar to findings in T2D, clustering analysis for PRED based on genotypic dataset identified six subtypes with distinct genetic score patterns and metabolic traits. Two of these subtypes exhibited a high risk of progressing to T2D^26^. These findings suggest that pathophysiological heterogeneity emerges before T2D onset, shaping in this way the course of the disease early on. An important open question for future research is the role of PRED cluster identification in predicting progression to diabetes and development of complications independent of diabetes progression. Other studies have examined disease progression in low- and high-risk PRED subgroups during the disease’s natural course and lifestyle intervention^27^. In contrast, the HEIST-DiC takes a holistic approach, providing a comprehensive clinical and metabolic assessment of diabetes-related complications.

The HEIST-DiC cohort provides a unique opportunity to assess individuals as usually seen in clinical practice. This study enables early identification of high-risk subgroups before complications manifest and investigates whether mechanisms driving (pre)diabetes onset contribute to the progression and development of complications. Most importantly, the cohort’s inclusion of individuals with long-term diabetes allows for the identification of factors distinguishing those with specific diabetes-associated complications from those without, beyond the presence and duration of diabetes. Leveraging the study’s intensive clinical-experimental characterization, HEIST-DiC enables the investigation of pathophysiological cellular mechanisms underlying different diabetes subgroups.

### Personalized intervention strategies for DM and its complications

Previous attempts to prevent or slow the progression of diabetes-associated complications have achieved only moderate success in reducing absolute risk for diabetes-associated complications in both type 1 diabetes (T1D)^28–30^ and T2D^31^. This limited success is likely due, in part, to the glucose-centered focus of these interventions, as well as the lack of consideration of DM subtypes. A post hoc analysis of longitudinal data from intensive lifestyle interventions in T2D, utilizing age at diabetes diagnosis and k-means clustering, identified a cluster with poor glucose control associated with increased cardiovascular risk after the intervention^32^. In a randomized controlled trial using a fasting-mimicking diet in a T2D subgroup from HEIST-DiC, assessing vulnerability to fasting required deep phenotyping and longitudinal observation to distinguish individuals who might benefit from the intervention from those at risk of adverse effects^33^. Data-driven cluster analysis demonstrated that individuals with SIRD benefit with better glycemic control from thiazolidinediones, while glycemic control in those with mild age-related diabetes is better controlled with sulfonylurea^18^. These findings emphasize that use of novel diabetes subtypes is justified only if they demonstrate clinical relevance, specifically through subtype-specific responses to stratified intervention strategies^34^. Focusing on pathophysiology-based therapeutic approaches is key to delivering personalized treatment in T2D, and subtype-specific randomized clinical trials will be critical in assessing the clinical relevance of these new DM subtypes^17,34^.

The HEIST-DiC cohort stands out for its comprehensive inclusion of individuals across the full spectrum of glucose metabolism—from normal glucose tolerance to prediabetes, recent-onset diabetes, and long-standing diabetes with and without complications. This breadth provides an unparalleled opportunity to address critical unanswered questions in diabetes research, including the validation and refinement of clustering methods, the stability of subtypes over time, and the identification of novel biomarkers and high-risk individuals. By enabling the stratification of participants into subtypes and focusing on their pathophysiological mechanisms, the HEIST-DiC cohort bridges the gap between large-scale epidemiological studies and clinical-experimental research. This approach will not only validate existing clustering methodologies but also drive the development of new frameworks for understanding the dynamic and heterogeneous nature of diabetes. Ultimately, the cohort’s design supports the testing of tailored therapeutic strategies and interventions, advancing the clinical relevance of subtype-specific approaches and paving the way for precision medicine in diabetes care^35^.

## Study design and methods

The Heist-DiC is an ongoing monocentric, prospective longitudinal observational study involving intensive metabolic and clinical phenotyping conducted annually for at least 11 years. Participants are recruited from the Clinic of Endocrinology, Diabetology, Metabolic Diseases and Clinical Chemistry (Internal Medicine 1) and are evaluated at the Study Center for Diabetes and Metabolism at the University Hospital of Heidelberg in Germany.

The aims of the HEIST-DiC cohort are to identify: (i) DM subtypes with different development patterns of diabetes-associated complications, (ii) predictors of diabetes-associated complications at early and late disease stages, (iii) pathophysiological mechanisms beyond hyperglycemia that contribute to the onset of diabetes-associated complications, (iv) novel nonclassical diabetes-associated complications, (v) personalized interventions for the prevention, improvement or remission of diabetes-associated complications.

The HEIST-DiC cohort is a hypothesis-generating study that will validate established clustering methods for diabetes classification, develop and apply new approaches – particularly for complications-specific clustering – and identify individuals from specific subtypes for targeted clinical-experimental studies.

### Composition of the cohort

To capture the various stages of disease development and progression, the study imposes no restrictions on disease stage or DM duration. Therefore, the study includes normal glucose tolerant individuals (NGT) and individuals with PRED, defined by oral glucose tolerance test (oGTT) and/or glycated hemoglobin (HbA1c) according to current ADA guidelines^36^, as well as individuals already diagnosed with DM (T1D or T2D) aged 18 to 85 years. Inclusion of older individuals in the cohort allows for a more accurate representation and assessment of age-related variability, while enabling identification of short- to medium-term trends that remain clinically relevant, particularly with respect to the mild age-related diabetes subtype^14,37^. Individuals with an initial HbA1c ≥ 9.5% are excluded, to minimize the risk of acute glycemic deterioration during study visits and its potential impact on diabetes-related complications. This also ensures participant safety and the accuracy of metabolic phenotyping under stable medical conditions; however, later inclusion is possible after appropriate treatment lowers their HbA1c to < 9.5%. Individuals with type 3 (e.g. pancreatogenic) or type 4 (gestational) diabetes, as well as pregnant women, are also excluded. A complete list of inclusion and exclusion criteria is detailed in Table 1.

**Table 1:** Inclusion and exclusion criteria.

Inclusion criteria	
- Age of 18–85 years - Diagnosis of type 1 and type 2 diabetes mellitus (DM) including maturity onset diabetes of the young (MODY) and latent autoimmune diabetes of the adult (LADA) based on current ADA recommendations - Individuals with prediabetes based on current ADA recommendations (individuals with impaired fasting glucose, impaired glucose tolerance or HbA1c 5.7% − 6.4%) - Glucose tolerant individuals based on current ADA recommendations (fasting plasma glucose < 100 mg/dL, 2-hour glucose level < 140 mg/dL, HbA1c < 5.7%)	
**Key exclusion criteria**	**Exclusion criteria for specific examinations**
- Secondary DM according to ADA criteria: type 3 B-H (e.g. pancreatogenic DM) or type 4 (gestational DM) - HbA1c ≥ 9.5% - Current pregnancy - Acute infections/fever - Immunosuppressive therapy - Severe psychiatric illness requiring treatment - Dependence on alcohol/other drugs - Severe heart, kidney, or liver disease: NYHA stage IV - Other causes of liver disease apart from non- alcoholic fatty liver disease i.e. autoimmune hepatitis, Morbus Wilson, Hemochromatosis, primary biliary cholangitis, primary sclerosing cholangitis - Severe peripheral artery disease (stage IV) - Non-diabetic glomerulopathy - Malignant cancer in the last 5 years - Infectious diseases i.e. hepatitis B, C, E, HIV - Autoimmune diseases requiring immunosuppressive therapy - Current participation in an intervention study - Anaemia or bone marrow disorders	- Magnetic resonance imaging: metallic implants (cochlear implants, clips, cardiac pacemaker or defibrillator, prosthetic valves), metallic fragments, large tattoos, claustrophobia, waist circumference > 135 cm, impaired renal function with eGFR < 65 ml/min, known allergy to contrast media - Euglycemic hyperinsulinemic clamp test: history of thrombosis or peripheral pulmonary artery embolism, laboratory values: ≤ 80% of the lower reference value for ferritin, iron, leukocytes, hemoglobin, haematocrit, erythrocytes, platelets, (residual) blood alcohol detection [‰]. - Bioelectrical impedance analysis: cardiac pacemaker or defibrillator - Pulmonary function testing: failure to correctly follow instructions - Tissue biopsies: effective anticoagulation therapy, platelet aggregation inhibitors > 100 mg acetylsalicylate, history of coagulation disorder, history of hypersensitivity to local anaesthetics

Abbreviations: ADA: American Diabetes Association^36^, NYHA: New York Heart Association, HIV: Human immunodeficiency virus, eGFR: estimated glomerular filtration rate.

Study participants are recruited through advertisements in local newspapers, public events, and information shared via the institutional website or health practitioners. Potential participants undergo a prescreening telephone interview to assess inclusion and exclusion criteria. Suitable candidates are then invited to the first study visit, which includes medical history, physical examination, blood withdrawal, questionnaires, and assessment of diabetes-associated complications. Decisions regarding participation in advanced examinations, such as the euglycemic hyperinsulinemic clamp test and tissue biopsies, are made and scheduled afterward. All participants provide written informed consent to the study protocol.

As of October 2023, the study has enrolled 552 participants, including 68 individuals with NGT, 119 with PRED, 83 with T1D and 282 with T2D. DM diagnosis and type are confirmed according to current ADA guidelines^36^ only for participants diagnosed at their first study visit, including those with latent autoimmune diabetes of the adult (LADA). No genetic analysis is conducted to assess for maturity onset diabetes of the young (MODY).

### Duration of the study

As of October 1 2023, all study participants reported here have completed the full study program at baseline and annually thereafter. Electrophysiological examination, by nerve conduction velocity (NCV), and quantitative sensory testing (QST) were not performed at year 3 and 5. Starting October 2023, participants undergo the complete study program at baseline and at years 4, 8, and 12, with a reduced study program in the years in between. A detailed list of examinations for each study visit is provided in Table 2. Recruitment is ongoing, making the precise dropout rate difficult to determine, particularly as participants may miss visits due to illness or scheduling problems. By beginning of October 2023, 19% of participants were lost to follow-up, with 11% of these being deceased (total mortality-rate 2%), while others cited reasons such as lack of time, transportation issues, or were simply inaccessible. Death cause was acute myocardial infarction (4 participants), hemorrhagic stroke (1 participant), respiratory failure due to fungal pneumonia (1 participant), urothelial carcinoma (1 participant), sarcoma (1 participant) and unknown (7 participants). 92% of the deceased participants were T2D individuals, predominantly male (69%) and 8 years older than the ongoing participants. The rest of the dropouts (non-deceased participants) were mostly females, 5 years older than the ongoing participants, with the group-distribution being comparable to the ongoing study participants. Nearly half (40%) of dropouts occurred in the first year following the baseline visit. Participants can pause their involvement for any reason and resume later. Most missed appointments were due to cancellations related to the Covid-19 pandemic, with visit 3 being the most affected (18% missed the visit, half due to Covid-19-related restrictions). Overall, the yearly follow-up visits (from visit 1 to visit 5), had an average response rate of 82%, including participants who attended or are still eligible to attend. As many participants are recruited through our university outpatient clinic, routine visits as part of standard care allow us to re-establish contact with those participants lost to follow-up, re-inform them about the study, and offer the opportunity to resume participation.

**Table 2:** Questionnaires and examinations for all visits as of October 2023.

		Follow-up		
	BL/FV	Every year	Every 2 years	Every 4 years
Demographics				
Age	X	X		
Sex	X	X		
Anthropometrics				
Body height & weight, body mass index	X	X		
Waist & hip circumference	X	X		
Diabetes				
Time of diagnosis	X	X		
Symptoms at time of diagnosis	X	X		
Diabetes treatment regime	X	X		
History of diabetic nephro-, retino-, neuropathy	X	X		
Cardiovascular complications ^1^	X	X		
Pulmonary complications	X	X		
Cerebrovascular complications ^1^	X	X		
Family history ^1^	X	X		
Currently or formerly overweight	X	X		
Diabetes risk test (for NGT and PRED)	X	X		
Oral glucose tolerance test (not for T1D) ^1^	X	X		
Personal health behaviour, life style				
Smoking ^1^	X	X		
Alcohol ^1^	X	X		
Diet ^1^	X	X		
Other diseases ^1^	X	X		
Medication	X	X		
Health-related quality of life				
SF-12, PHQ ^2^	X			X
Cardiovascular examinations				
Vascular status	X	X		
Electrocardiogram	X	X		
Sonography neck vessels & abdominal aorta ^2^	X		X	X
Ankle–brachial index ^2^	X		X	X
24 h-blood pressure measurement ^2^	X		X	X
6-minute walk test ^2, 3, 4^	X		X	X
Neurological examinations				
NDS, NSS, SAS, foot inspection	X	X		
MNSI ^5^	X			X
Foot & Hand nerve conduction velocity ^2, 6, 7^	X			X
Transcutaneous electrical nerve fiber stimulation^7^				
Quantitative sensory testing ^2^	X			X
Heart rate variability ^2^	X			X
Manual strength ^8^	X	X		
Pegboard ^2, 8^	X			X
Hepatic examinations				
Elastography ^2, 9, 10,^	X			X
Sonography liver ^2^	X		X	X
Renal examination				
Sonography kidney ^2^	X		X	X
Ophthalmological examination				
Fundoscopy	X	X		
Pulmonary examination				
Spirometry, DL_CO_, body plethysmography ^2^	X			X
Other examinations				
Bioelectrical impedance analysis	X	X		
Skin autofluorescence ^2^	X			X

All examinations and questionnaires are performed at the initial baseline (BL) and final visit (FV) and are repeated every year (visits 1–10) or only additionally every two (visits 2, 4, 6, 8, 10) or four (visits 4, 8) years according to the current study protocol of October 2023. Previous changes to the study protocol: ^**(1)**^ Anamnesis for cardio- and cerebrovascular complications, Family history, Smocking, Alcohol, Diet and other diseases now yearly and not only at BL, expanded questionnaires for Family history and Alcohol consumption, oGTT for T2D starting from February 2023; ^**(2)**^ Examinations were reduced from yearly or biyearly (nerve conduction velocity & quantitative sensory testing) to every second or forth year from October 2023; ^**(3)**^ 6-minute walk test added from December 2016; ^**(4)**^ 6-minute walk test discontinued between January 2021 and April 2023; ^**(5)**^ Michigan neuropathy screening instrument (MNSI) for visits BL (starting proband 447), 4, and 6; added from September 2020 ^**(6)**^ Hand nerve conduction velocity for visit 4 and 6 added from February 2021; ^**(7)**^ transcutaneous electrical nerve fiber stimulation added from June 2023; ^**(8)**^ Manual strength & Pegboard added from November 2017; ^**(9)**^ liver stiffness (elastography) added from November 2016; ^**(10)**^ controlled attenuation parameter (elastography) added from August 2017.

Abbreviations: NGT: normal glucose tolerant individuals, PRED: individuals with prediabetes, T1D: individuals with type1 diabetes, SF-12: 12-item short-form health survey, PHQ: patient health questionnaire, NDS: neuropathy disability score, NSS: neuropathy symptom score, SAS: symptom assessment score, DL_CO_: diffusing capacity of the lung for carbon monoxide.

### Measurements

Demographic data and diabetes-specific medical history, including current medication, are recorded at baseline and updated during each follow-up visit (Table 2). Clinical and metabolic variables are also documented at both baseline and follow-up visits (Tables 3 and 4).

To assess chronic kidney disease (CKD), the study utilizes albuminuria, estimated glomerular filtration rate (eGFR), and kidney ultrasound^33,38^. Distal sensorimotor polyneuropathy (DSPN) is evaluated using the neuropathy symptoms score (NSS), neuropathy disability score (NDS), Purdue pegboard test, NCV, transcutaneous electrical nerve fiber stimulation, QST in accordance with the DFNS (German Research Network on Neuropathic Pain) protocol, and high-resolution magnetic resonance neurography (MRN)^39–46^. Heart rate variability (HRV) is measured to assess cardiovascular autonomic neuropathy (CAN)^12^. Anamnestic assessment of CAN is performed using the SAS questionnaire^47^. Ophthalmological assessment include a funduscopic examination of the undilated pupil for DR^48^. Diabetes related restrictive lung diseases (RLD) is evaluated through the 6-minute walk test, spirometry, body plethysmography, and carbon monoxide-based diffusing capacity measurements (DL_CO_)^49,50^. Transient elastography and hepatic ultrasound are conducted to evaluate MASLD and liver fibrosis^51^. The ankle-brachial index and carotid intima-media thickness are used to examine peripheral atherosclerosis^52^. Hand grip strength and bioelectrical impedance analysis (BIA) are utilized to assess muscle strength and body composition^52,53^.

OGTT and euglycemic hyperinsulinemic clamp test are conducted to assess beta-cell function and insulin sensitivity^54^. Hepatic insulin sensitivity is measured through the co-infusion of [6,6-^2^H_2_]glucose^55^. Additionally, energy expenditure and substrate oxidation during fasting and hyperinsulinemia are evaluated using indirect calorimetry^56^.

At each visit, whole-blood, erythrocytes, leucocytes, plasma (EDTA), serum and urine samples are stored at −80 °C for biomarker analysis^57,58^. Beginning in 2022, skeletal muscle, subcutaneous adipose tissue, and skin samples can also be collected for future analyses. The collected biospecimens support a broad spectrum of molecular and cellular analyses, including biomarker discovery, tissue-specific investigations of insulin resistance, inter-tissue communication and metabolic dysregulation, as well as mechanistic studies using patient-derived specimens.

Health-related quality of life and physical health are assessed using the 12-item short-form health survey (SF-12), while somatic and depression symptoms are evaluated with the patient health questionnaire (PHQ)^59,60^. A detailed overview of the measurements is provided in Table 2, and the experimental protocols are described in Supplementary file 1.

### Definition of diabetes-associated complications

CKD was defined by an increased urinary albumin-to-creatinine ratio (uACR ≥ 30 mg/g) and/or a decreased eGFR (< 60 ml/min/1.73 m^2^), following the Kidney Disease: Improving Global Outcomes (KDIGO) guidelines for DM management of CKD^61^.

DSPN was defined by the presence of either at least moderate neuropathic deficits (NDS ≥ 6) or the combination of mild deficits with moderate neuropathic symptoms (NSS ≥ 5 together with NDS 3–5)^62^, or according to the Toronto consensus definition of confirmed DSPN: abnormal results of the nerve conduction study of the sural nerve and additional at least one abnormal parameter from nerve conduction studies of the common peroneal or tibial nerve (parameters below the 2.5 percentile values of the cohort) together with an NSS and/or NDS of ≥ 3^41,58,63,64^. Cases with bilaterally non-recordable sural nerve were assessed as having abnormal sensory function and values were calculated as lowest value of the cohort.

CAN was defined by two pathological values for age-dependent beat-to-beat variation with deep breathing (E/I-quotient), 30/15 heart rate ratio with standing, or orthostatic hypotension (systolic or diastolic change below the 2.5 percentile values of the cohort)^12,65,66^.

DR was defined by criteria indicating at least stage I of retinopathy based on funduscopic examination of the undilated pupil, as previously reported^67^. In this study, we do not analyze non-proliferative or proliferative DR.

Diabetes related RLD was defined by reduced forced vital capacity (FVC < 80%), reduced total lung capacity measured with body plethysmography (TLC-B < 80%), or reduced single-breath diffusing capacity of the lung for carbon monoxide (DL_CO_ < 80%), in the presence of a normal forced expiratory volume in 1 s adjusted to vital capacity (FEV_1_/VC > 70%), as previously reported^49,68^.

MASLD was defined by an increased controlled attenuation parameter (CAP) (M-probe: CAP ≥ 248 dB/m, XL-probe: CAP ≥ 302 dB/m) measured by transient elastography. Liver fibrosis was defined by increased liver stiffness (≥ 8 kPa) measured by transient elastography^41^.

## Discussion

### What has it found? Key findings and publications

#### Baseline characteristics

Baseline characteristics of the cohort recruited between September 2016 and October 2023 are presented in Tables 3 and 4. The percentage of individuals diagnosed with early-onset T2D (diagnosis at age < 45 years) in our cohort aligns with findings from previous studies based on U.S. registry data (Table 3)^69^. Additionally, the prevalence of PRED at an early age (< 45 years) is consistent with global prevalence rates of PRED (Table 3)^70^. In our cohort, the percentage of males with T2D is higher than that of males with T1D, reflecting trends seen in a previously reported German cohort of individuals with recent-onset T1D and T2D (Table 3)^71^. Overall, our cohort includes more male participants than female participants with PRED, T1D, and T2D. The increased proportion of males in the pathological DM groups aligns with findings from population-based studies^72,73^ and likely reflects the known underrepresentation of females in clinical research^74^. There is a higher percentage of female participants than male participants in the NGT group (Table 3), potentially attributed to the greater motivation of healthy women to participate in time-consuming examinations. To address this limitation and ensure a more balanced representation, we are planning to recruit a higher proportion of male participants in the future through public events, as well as through information shared on the institutional website and by healthcare practitioners. For the analysis of different subtypes, study participants will be matched for sex, age at diabetes diagnosis, BMI, glycemia, and homoeostasis model estimates calculated using c-peptide (HOMA-IR).

**Table 3:** Baseline characteristics in individuals with NGT, PRED, T1D and T2D.

	NGT	PRED	T1D	T2D
Age <20 20–24 25–29 30–34 35–39 40–44 45–49 50–54 55–59 60–64 65–69 70–74 75–80 >80	0 2.9 5.9 4.4 5.9 8.8 13.2 10.3 22.1 10.3 10.3 4.4 1.5 0	0 0 0 2.5 3.4 3.4 9.2 20.2 19.3 18.5 15.1 5.0 2.5 0.8	3.6 8.4 10.8 9.6 9.6 3.6 4.8 7.2 15.7 9.6 8.4 4.8 2.4 1.2	0 0 0.4 0 2.8 2.8 6.0 9.6 11.0 19.5 19.5 18.8 9.2 0.4
Sex Male Female	30.9 69.1	52.1 47.9	50.6 49.4	59.9 40.1
**Diabetes duration** <1 1–5 6–10 11–15 16–20 21–25 26–30 31–35 36–40 41–45 46–50 51–55 56–60 61–65 >66	NA NA NA NA NA NA NA NA NA NA NA NA NA NA NA	NA NA NA NA NA NA NA NA NA NA NA NA NA NA NA	10.8 13.3 4.8 13.3 10.8 10.8 6.0 9.6 6.0 2.4 4.8 2.4 2.4 1.2 1.2	12.1 24.1 20.6 16.7 16.0 5.3 3.2 1.1 0.7 0.4 0 0 0 0 0
**Family history of diabetes** Siblings Father Mother Grandparents (father side) Grandparents (mother side)	13.2 16.2 25.0 4.4 13.2	11.8 19.3 23.5 6.7 15.1	9.6 6.0 12.1 13.3 21.7	24.5 29.1 32.3 9.9 14.2
**Other diseases** Hypertension History of myocardial infarction Coronary heart disease Current smoking Lung diseases Diabetic retinopathy Diabetic nephropathy Diabetic neuropathy	17.7 0 2.9 10.3 16.2 0 0 0	43.7 5.9 7.6 8.4 16.0 0 0 1.7	49.4 6.0 7.2 16.9 9.6 6.0 21.7 28.9	77.0 7.1 16.7 12.8 19.9 11.0 8.9 47.2
**Glucose lowering therapy** Insulin (short acting) Insulin (long acting) Insulin (mixed) Metformin Acarbose Sulfonylurea Glinide Dipeptidyl-peptidase-4 inhibitors PPAR agonists GLP-1 agonists SGLT-2 inhibitors	0 0 0 2.9 0 0 0 0 0 0 0	0 0 0 0.8 0 0 0 0 0 0 0	97.6 72.3 1.2 4.8 0 0 0 0 0 0 2.4	18.8 25.9 1.1 62.4 0.4 4.3 1.1 20.6 0.7 6.4 16.7
**Antihypertensive therapy** Blockers of the renin-angiotensin system Beta blockers Calcium channel blockers Alpha1 inhibitors Alpha2 agonists Diuretics	13.2 7.4 4.4 1.5 0 4.4	35.3 19.3 13.5 3.4 0 11.8	33.7 18.1 14.5 4.8 0 12.1	61.0 34.4 22.7 7.5 3.6 33.0
**Other therapies** Acetylsalicylic acid Statins Clopidogrel Tricyclic antidepressants Anticonvulsants Inhaled anticholinergics Inhaled beta2 agonists Inhaled corticoids L-Thyroxin	8.8 5.9 0 0 1.5 1.5 2.9 2.9 16.2	9.2 16.8 0 1.7 0.8 1.7 6.7 4.2 24.4	19.3 27.7 0 1.2 3.6 0 2.4 1.2 24.1	30.1 45.0 2.1 4.3 9.9 1.4 4.3 2.5 24.5
**Cardiovascular risk (ESC-Score 2)** low to moderate high very high	51.9 36.5 11.5	31.2 45.0 23.9	27.3 34.1 38.6	14.3 50.8 34.9

Demographics of individuals with normal glucose tolerance (NGT), prediabetes (PRED), type 1 diabetes (T1D) and type 2 diabetes (T2D) at baseline by 10/2023. Percentages per individual groups.

Abbreviations: PPAR: peroxisome proliferator-activated receptor, GLP-1: glucagon-like peptide 1, SGLT-2: sodium-glucose cotransporter 2, ESC: European Society of Cardiology.

Family history of DM was more frequent in the T2D group (Table 3). The cohort has not been screened for genetic defects that could indicate MODY. Diagnosis of DM and its respective type is confirmed only for participants diagnosed with DM at the first study visit, which means individuals with MODY may still be included in the cohort.

Most participants with T1D (76%) had a DM duration exceeding 5 years (Table 3), and therefore a negligible residual beta-cell function (Table 4, Panel b). As expected, participants with T2D exhibited low insulin sensitivity and high whole-body and adipose tissue insulin resistance (Table 4, Panel b). In our cohort glycemic control at baseline appears to be slightly better in T2D participants compared to those with T1D (Table 4, Panel b). All but one individual with T1D were treated with insulin and only 5% with metformin, which was the most common medication in individuals with T2D (62%). Notably metformin was also taken by 2 participants with NGT (one with a history of polycystic ovary syndrome and one diagnosed with insulin resistance) and 1 with PRED (indication of metformin therapy unknown), Additionally, 2% of participants with T1D were treated with SGLT-2 inhibitors (Table 3).

**Table 4:** Baseline anthropometric and metabolic characteristics in individuals with (a) NGT/PRED, and (b) T1D/T2D.

Panel a	NGT				PRED				
	*N*	Mean ± SD	Median	LQ/UQ	*N*	Mean ± SD	Median	LQ/UQ	
Age (years)	68	51.4 ± 13.1	54.0	43.8/60.3	119	57.2 ± 9.9	56.0	51.0/64.0	
Body mass index (kg/m2)	68	26.3 ± 4.6	25.6	22.9/28.8	119	29.7 ± 5.9	28.6	25.5/33.0	
Waist-hip-ratio	68	0.9 ± 0.1	0.9	0.8/0.9	119	0.9 ± 0.1	0.9	0.9/1.0	
Glucose (mg/dL)	67	88.4 ± 6.4	89.0	85.0/92.0	119	101.8 ± 10.7	100.0	95.0/106.5	
Hemoglobin A1c (%)	68	5.2 ± 0.3	5.3	5.1/5.4	119	5.6 ± 0.3	5.7	5.4/5.8	
C-peptide (ng/mL)	61	2.0 ± 0.7	1.7	1.5/2.2	102	2.7 ± 1.3	2.3	1.7/3.4	
HOMA-IR	63	2.0 ± 1.1	1.7	1.3/2.5	103	3.1 ± 2.1	2.5	1.7/3.8	
AUC-Glucose-oGTT (total)	67	12719.6 ± 2470.4	12570.0	11205.0/14235.0	116	16493.5 ± 3054.7	16350.0	14422.5/18780.0	
Matsuda ins. sens. index	62	7.1 ± 3.7	6.9	4.3/8.8	101	4.4 ± 3.0	3.9	2.4/5.1	
FFA (µmol/L)	38	581.1 ± 254.3	596.6	363.9/691.6	83	637.9 ± 253.1	634.0	423.2/758.2	
Adipo-IR (mmol*pmol/L)	38	33.8 ± 25.5	25.3	16.1/40.1	83	56.4 ± 45.2	40.5	23.1/77.3	
Total cholesterol (mg/dL)	68	204.2 ± 43.3	203.0	172.0/230.3	119	205.6 ± 38.0	208.0	176.0/228.0	
HDL cholesterol (mg/dL)	68	63.0 ± 16.7	61.0	49.0/73.0	119	57.0 ± 16.6	53.0	45.0/64.5	
LDL cholesterol (mg/dL)	68	122.5 ± 37.6	116.5	93.0/147.3	119	123.4 ± 33.5	123.0	100.0/144.0	
Triglycerides (mg/dL)	68	94.1 ± 41.0	80.5	63.0/119.8	119	125.7 ± 65.8	108.0	78.5/164.5	
ASAT (U/L)	68	22.3 ± 6.9	21.0	17.8/26.3	119	24.1 ± 8.0	23.0	19.0/27.0	
ALAT (U/L)	68	21.6 ± 9.6	19.0	15.8/25.3	119	27.9 ± 15.1	24.0	18.0/32.5	
GGT (U/L)	68	20.9 ± 20.5	16.5	11.0/22.0	119	34.8 ± 83.6	23.0	14.5/32.0	
FIB-4	68	1.2 ± 0.7	1.1	0.6/1.4	119	1.2 ± 0.5	1.1	0.8/1.4	
MASLDS	66	−2.3 ± 1.2	−2.1	−3.2/−1.4	118	−2.1 ± 1.1	−2.1	−2.8/−1.3	
FLI	68	35.7 ± 26.8	29.5	11.4/60.0	119	58.1 ± 31.8	60.9	28.3/90.7	
NTproBNP (ng/L)	63	78.7 ± 59.3	56.0	32.5/118.0	114	80.9 ± 74.9	62.0	32.0/104.0	
Lp(a) (mg/dL)	66	24.9 ± 26.6	9.9	9.9/26.8	112	26.4 ± 37.2	10.0	9.9/27.2	
hsTNT (mg/mL)	63	5.9 ± 3.0	5.0	4.0/7.0	114	7.3 ± 4.8	7.0	5.0/9.0	
hsCRP (mg/L)	65	2.2 ± 3.5	0.6	0.4/1.9	114	2.9 ± 4.0	1.2	0.6/3.0	
BP syst (mmHg)	67	128.6 ± 20.1	125.5	113.3/138.8	119	134.9 ± 16.7	134.5	121.3/145.3	
BP dia (mmHg)	67	82.0 ± 11.7	79.5	73.8/89.3	119	84.5 ± 10.1	84.5	76.5/91.0	
ESC-Score2	52	5.3 ± 4.9	4.0	2.8/7.0	109	7.1 ± 4.6	6.0	4.0/10.0	
***Panel b***	**T1D**				**T2D**				
	**N**	**Mean ± SD**	**Median**	**LQ/UQ**	**N**	**Mean ± SD**	**Median**	**LQ/UQ**	
Age (years)	83	46.5 ± 17.6	49.0	30.0/61.0	282	62.4 ± 10.2	64.0	56.0/70.0	
Body mass index (kg/m2)	83	25.9 ± 4.5	25.3	23.3/28.4	282	31.0 ± 6.0	29.9	26.3/34.3	
Waist-hip-ratio	81	0.9 ± 0.1	0.9	0.8/1.0	281	1.0 ± 0.1	1.0	0.9/1.1	
Glucose (mg/dL)	83	177.7 ± 70.7	167.0	128.5/212.5	282	151.8 ± 46.9	143.5	122.0/170.0	
Hemoglobin A1c (%)	83	7.7 ± 1.5	7.5	6.6/8.7	282	7.2 ± 1.3	7.1	6.3/7.8	
C-peptide (ng/mL)	80	0.4 ± 0.6	0.1	0.1/0.4	220	3.1 ± 1.8	2.8	1.9/4.1	
HOMA-IR^1^	NA	NA	NA	NA	188	5.7 ± 8.2	4.3	2.5/6.5	
AUC-Glucose- oGTT (total)	NA	NA	NA	NA	12	21370.0 ± 5848.7	20010.0	18075.0/24967.5	
Matsuda ins. sens. index	0	0	0	0	12	2.9 ± 2.2	1.9	1.7/3.4	
FFA (µmol/L)	76	630.2 ± 391.9	588.7	314.5/883.9	124	684.2 ± 276.9	632.1	487.7/887.7	
Adipo-IR (mmol*pmol/L) ^1^	NA	NA	NA	NA	88	66.1 ± 54.0	47.8	28.3/88.6	
Total cholesterol (mg/dL)	83	194.3 ± 45.5	194.0	157.0/233.0	282	190.0 ± 48.6	183.5	157.0/221.0	
HDL cholesterol (mg/dL)	83	65.4 ± 18.9	65.	51.5/79.0	282	50.3 ± 15.7	47.0	39.0/57.0	
LDL cholesterol (mg/dL)	82	108.0 ± 38.0	101.5	79.0/134.0	261	105.5 ± 39.8	100.0	76.0/131.0	
Triglycerides (mg/dL)	83	103.4 ± 68.9	76.0	61.5/116.5	282	183.6 ± 167.1	136.5	102.0/205.8	
ASAT (U/L)	83	23.4 ± 8.4	22.0	18.0/27.0	282	26.2 ± 14.8	23.0	19.0/29.0	
ALAT (U/L)	83	23.0 ± 11.9	20.0	15.0/27.0	282	30.9 ± 20.8	25.5	19.0/36.0	
GGT (U/L)	83	22.1 ± 17.7	17.0	11.5/24.5	282	43.4 ± 62.3	28.0	18.0/45.0	
FIB-4	83	1.0 ± 0.5	0.9	0.5/1.3	282	1.4 ± 1.0	1.3	0.9/1.7	
MASLDS	81	−1.5 ± 1.3	−1.6	−2.6/−0.6	278	−0.5 ± 1.3	−0.4	−1.2/0.4	
FLI	81	35.3 ± 31.2	22.2	8.1/61.7	281	72.4 ± 26.2	81.5	54.8/95.0	
NTproBNP (ng/L)	75	120.3 ± 120.4	80.5	36.0/160.8	255	144.0 ± 325.1	68.0	35.0/137.5	
Lp(a) (mg/dL)	82	28.0 ± 34.0	10.0	9.9/26.2	246	27.3 ± 33.7	10.0	9.9/29.6	
hsTNT (mg/mL)	76	7.9 ± 6.0	6.0	4.0/8.9	255	10.8 ± 8.9	9.0	6.0/13.0	
hsCRP (mg/L)	82	2.86 ± 5.17	1.2	0.5/3.2	276	3.0 ± 3.8	1.7	0.7/3.6	
BP syst (mmHg)	82	135.2 ± 20.5	132.5	120.0/146.8	278	141.6 ± 17.6	138.0	128.5/152.9	
BP dia (mmHg)	82	81.6 ± 12.2	80.8	72.8/87.9	278	85.4 ± 9.6	85.0	79.5/91.0	
ESC-Score2	44	7.9 ± 4.9	7.0	4.0/11.3	258	9.9 ± 5.7	9.0	5.0/13.0	

Number (N) of individuals with normal glucose tolerance (NGT), prediabetes (PRED), type 1 diabetes (T1D) or type 2 diabetes (T2D) for the respective parameter, mean with standard deviation (SD) and median with lower (LQ) and upper quartile (UQ). ^1^ only individuals without insulin therapy.

Abbreviations: HOMA-IR: homeostatic model assessment of insulin resistance, AUC: area under the curve, oGTT: oral glucose tolerance test, ins sens.: insulin sensitivity, FFA: free fatty acids, Adipo-IR: adipose tissue insulin resistance index, HDL: high-density lipoprotein, LDL: low density lipoprotein, ASAT: aspartate aminotransferase, ALAT: alanine aminotransferase, GGT: gamma glutamyl transferase, FIB-4: fibrosis-4 index; MASLDS: metabolic dysfunction-associated steatotic liver disease score; FLI: fatty-liver-index; NTproBNP: n-terminal pro B-type natriuretic peptide, Lp(a): lipoprotein (a), hsTNT: high-sensitivity troponin T, hsCRP: high-sensitivity c-reactive protein, BP: blood pressure, syst: systolic, diast: diastolic, ESC: European Society of Cardiology.

While our cohort is not population-based and primarily focuses on diabetes-associated complications and distinct pathophysiological mechanisms, the mean age and body mass index (BMI) of participants align closely with other German and European cohorts (Table 4, Panel a and Panel b)^71,75–77^. As anticipated, participants with T1D are younger and leaner compared to those with T2D (Table 4, Panel b) and PRED. However, T1D participants exhibit similar age and BMI to those with NGT (Table 4, Panel a).

Participants in the T2D group exhibited a high prevalence of cardiovascular disease at baseline (Table 3). Hypertension was prevalent among T2D participants, with 77% reporting the condition, corresponding to a higher usage of antihypertensive therapy (69%) in this group compared to T1D (43%). Despite higher use of antihypertensive medication, the mean blood pressure values are higher in T2D participants than in those with T1D (Table 4, Panel b). Among PRED participants, 44% reported hypertension, with 40% receiving antihypertensive therapy. In the NGT group, 18% reported hypertension, and a similar percentage was on hypertensive therapy at baseline (Table 3). Statin use at baseline was reported by 6% of participants with NGT, 17% with PRED, 28% with T1D, and 45% with T2D (Table 3). While overall statin use was low across all groups, the undertreatment was particularly notable in T1D and T2D participants, with 73% of T1D participants and 86% of T2D participants classified as having high to very high cardiovascular risk according to the ESC-Score 2, independent of DM as a risk-factor (Table 3). Although low-density lipoprotein (LDL) levels for both T1D and T2D participants (Table 4, Panel b) are lower than in individuals with NGT and PRED (Table 4, Panel a), this poor utilization of statins is still reflected in the small proportion of participants achieving LDL-cholesterol goal levels, with only 17% of T1D and 30% of T2D participants meeting these targets.

Liver transaminases and lipoprotein (a) [Lp(a)] levels were comparable across all study groups (Table 4, Panel a and Panel b). However, levels of high-sensitivity C-reactive protein (hsCRP) were elevated in all groups except the NGT group, indicating a possible inflammatory response in participants with DM and PRED. N-terminal pro–B-type natriuretic peptide (proBNP) was elevated above the upper limit (125 ng/L < 71 year old; 450 ng/L for ≥ 71 year old) in 35% of T1D and 22% of T2D, and levels of high-sensitivity cardiac troponin T (hsTNT) were elevated above the upper limit (> 14 ng/L) in 14% of T1D and 27% of T2D (Table 4, Panel b), as markers indicating cardiac dysfunction and damage of heart muscle, respectively.

**Fig. 1:**
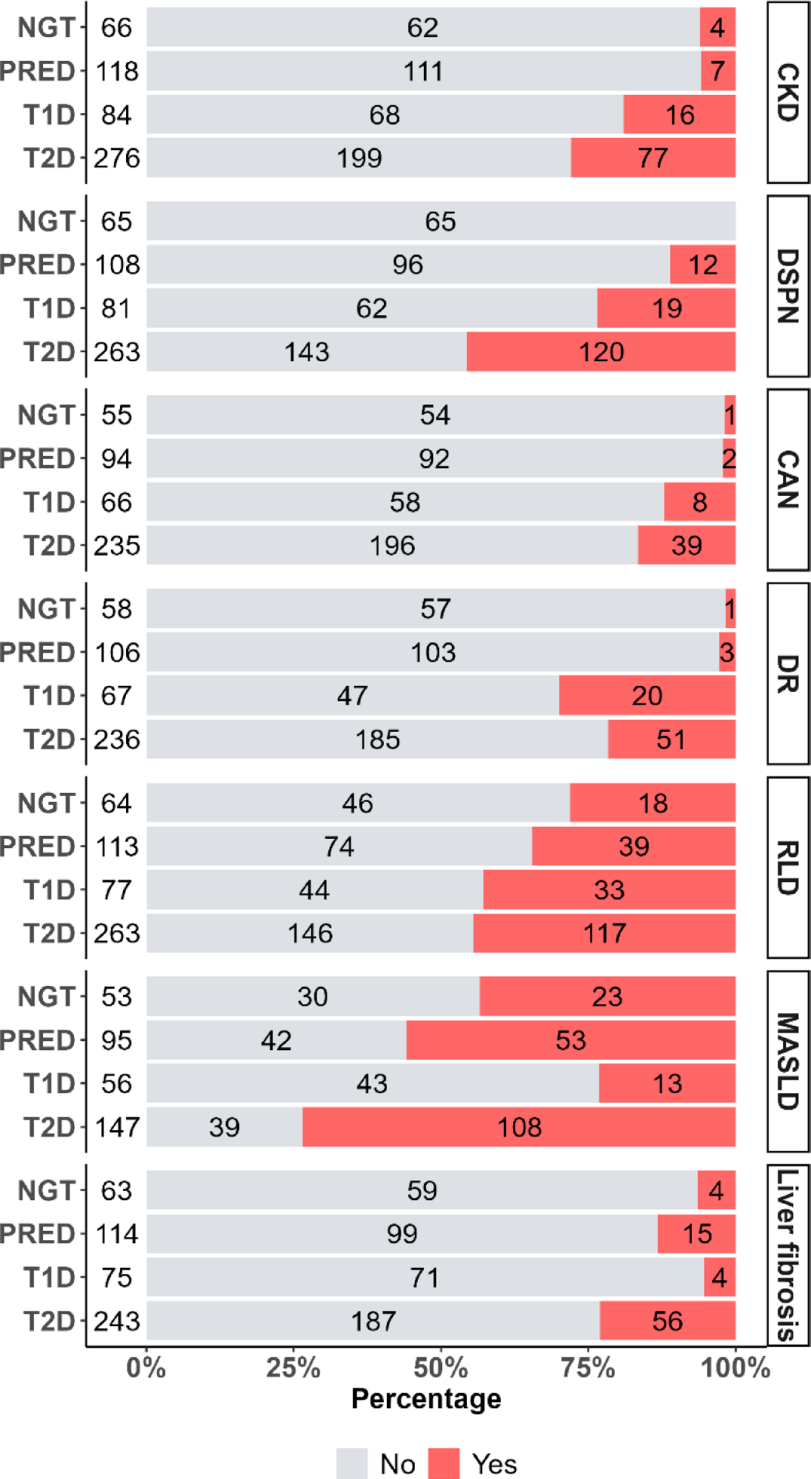
Diabetes-associated complications in individuals with NGT, PRED, T1D and T2D. Percentage (x-axis) and total numbers (in bars) of individuals without (grey) and with (red) respective diabetes-associated complications. CKD: chronic kidney disease, DSPN: diabetic sensorimotor peripheral neuropathy, CAN: cardiovascular autonomic neuropathy, DR: diabetic retinopathy, RLD: diabetes related restrictive lung disease, MASLD: metabolic dysfunction-associated steatotic liver disease.

The primary focus of this cohort is the assessment of diabetes-associated complications, with findings summarized in Fig. 1; Table 5. At baseline, CKD was identified in 19% of individuals with T1D and 28% of those with T2D (Fig. 1). Additionally, uACR levels were elevated in both the T1D and T2D group compared to the NGT and PRED group, with T2D participants showing higher uACR levels than those of the T1D group (Table 5 Panel a and Panel b). The increased prevalence of CKD in T2D is supported by a greater usage of renin-angiotensin system blockers (Table 3). According to previous European cohort studies, CKD is more common in T1D than in T2D^78,79^. The higher prevalence of CKD in T2D within our cohort may be attributed to a greater number of T2D participants in older age groups despite longer diabetes duration, whereas the T1D group consists mainly of younger individuals (Table 2). This is partly due to the initial active recruitment of individuals with DM and established diabetic complications, such as increased albuminuria. A few participants from the non-DM groups also met criteria for diagnosing CKD at baseline, specifically four from the NGT group and seven from the PRED group (Fig. 1). This may be related to other underlying conditions contributing to nephropathy, such as hypertension or undiagnosed renal diseases. In terms of DSPN, baseline prevalence was found to be 11% in PRED participants, 23% in T1D, and 46% in T2D (Fig. 1). The higher prevalence of DSPN in T2D aligns with previous intervention and cohort studies, highlighting the increasing burden of DSPN in older populations^80–82^. A systematic review on DSPN prevalence in adults with PRED indicated that 72% of studies reported a prevalence of ≥ 10%, consistent with our findings^83^. CAN was identified in 11% of T1D and 17% of T2D participants (Fig. 1), with only 2% of PRED participants and 2% of NGT participants meeting the criteria for CAN at baseline. The observed prevalence of CAN in PRED in our cohort is notably lower than previously reported estimates^65,84^, likely due to variability in the definitions and methodologies used in earlier studies^84^. For DR, our cohort reported a higher prevalence in T1D (30%) compared to T2D (22%), consistent with findings from previous studies^85,86^, with only one case identified in the NGT group and three cases in the PRED group. Diabetes related RLD phenotype was found in 28% of NGT participants, 35% of PRED participants, 43% of T1D participants, and 44% of T2D participants (Fig. 1). These numbers exceed those previously reported^49^, indicating a potential underdiagnosis of diabetes related RLD that warrants further investigation in future studies, especially in terms of treatment responses compared to non-diabetic cases with RLD^50^. The prevalence of MASLD was reported as 43% in the NGT group, 56% in the PRED group, 23% in the T1D group, and 73% in the T2D group, whereas the prevalence for liver fibrosis was 6% in NGT, 13% in PRED, 5% in T1D and 23% in T2D (Fig. 1). The higher prevalence in PRED and T2D was also reflected by elevated liver enzymes and triglycerides in the PRED and T2D groups, with T2D expressing the highest levels (Table 4 Panel a and Panel b). While the fatty-liver-index (FLI), as an index of MASLD, follows the same pattern, the fibrosis indices - fibrosis-4 index (FIB-4) and metabolic dysfunction-associated steatotic liver disease fibrosis score (MASLDS) - were only elevated in T2D (Table 4 Panel a and Panel b). The high prevalence of MASLD in NGT, PRED and T2D is likely due to high BMI in our cohort (Table 4 Panel a and Panel b), since BMI was previously reported to be the strongest covariate in predicting MASLD when using transient elastography^87^. The prevalence of MASLD in T1D in our cohort is slightly lower than in previous reported studies^88,89^. The higher prevalence for liver fibrosis, measured by elastography, in individuals with PRED and T2D and the fibrosis indices indicating a higher risk for advanced liver fibrosis for T2D, highlight the important role of impaired glucose metabolism in progression to liver fibrosis.

**Table 5:** Parameters of diabetes-associated complications in individuals with (a) NGT/PRED, and (b) T1D/T2D.

Panel a	NGT				PRED				
	*N*	Mean ± SD	Median	LQ/UQ	*N*	Mean ± SD	Median	LQ/UQ	
CKD									
Creatinine (mg/dL)	68	0.8 ± 0.2	0.8	0.7/0.9	119	0.8 ± 0.2	0.8	0.7/0.9	
eGFR (CKD-EPI, ml/min/1.73m^2^)	68	95.2 ± 14.1	95.2	86.0/103.3	119	92.7 ± 13.0	93.9	84.0/101.1	
uACR (mg/g)	67	10.1 ± 12.8	6.1	4.5/10.1	118	9.3 ± 16.2	5.1	3.4/8.8	
DSPN^1^									
NDS	68	0.5 ± 1.0	0.0	0.0/1.0	116	1.4 ± 1.7	1.0	0.0/2.0	
NSS	67	0.6 ± 1.8	0.0	0.0/0.0	114	1.4 ± 2.8	0.0	0.0/0.0	
NCV sural nerve (m/s)	66	49.0 ± 5.2	48.0	45.9/52.0	101	43.4 ± 13.3	47.0	41.0/51.0	
SNAP sural nerve (µV)	66	10.4 ± 6.2	9.0	5.8/13.3	101	8.5 ± 7.5	7.3	3.7/11.3	
NCV peroneal nerve (m/s)	67	45.7 ± 4.6	46.0	43.0/47.0	106	44.8 ± 4.9	45.7	41.2/48.0	
CNAP peroneal nerve (mV)	67	6.9 ± 2.9	6.9	5.1/8.4	106	7.3 ± 3.3	7.1	5.5/9.4	
DML peroneal nerve (ms)	67	4.1 ± 0.7	4.0	3.6/4.6	106	4.2 ± 0.9	4.0	3.8/4.4	
NCV tibial nerve (m/s)	55	46.2 ± 4.8	45.0	43.3/47.7	93	44.2 ± 4.7	44.0	41.0/47.0	
CNAP tibial nerve (mV)	55	19.0 ± 7.4	18.9	14.2/22.3	93	16.1 ± 7.7	15.8	11.0/20.3	
DML tibial nerve (ms)	55	3.8 ± 0.6	3.6	3.4/4.05	94	3.9 ± 1.0	3.7	3.2/4.2	
CAN									
E/I-quotient	66	1.3 ± 0.2	1.2	1.1/1.3	109	1.2 ± 0.1	1.2	1.1/1.3	
30/15-quotient	66	1.3 ± 0.3	1.3	1.1/1.4	108	1.2 ± 0.4	1.2	1.1/1.3	
Orthostasis delta systolic	56	−0.5 ± 8.8	−1.5	−3.5/1.1	93	0.7 ± 10.8	1.0	−5.5/5.3	
Orthostasis delta diastolic	56	−3.9 ± 8.2	−3.9	−5.6/−1.2	93	−2.7 ± 7.6	−2.5	−5.0/0.0	
Diabetes related RLD									
Vital capacity (%)	67	106.9 ± 15.9	107.0	96.0/118.5	116	105.1 ± 13.4	104.0	95.0/114.3	
Tiffenau-Index (%)	67	96.9 ± 8.0	97.0	92.5/102.0	117	95.9 ± 9.6	96.0	91.0/102.0	
TLC body plethysmography (%)	67	109.0 ± 17.0	107.0	97.5/120.0	117	105.0 ± 14.6	102.0	94.0/115.0	
Diffusing capacity for CO2 (%)	65	88.3 ± 17.3	87.0	79.0/99.0	117	88.0 ± 17.2	87.0	76.0/99.0	
6-min walk-test (m)	53	607.8 ± 67.5	609.0	559.1/648.0	92	575.0 ± 75.4	576.4	544.5/615.1	
MASLD/Liver fibrosis									
Liver stiffness (kPa)	64	5.5 ± 3.2	5.1	4.2/6.0	113	6.1 ± 2.6	5.5	4.6/7.2	
CAP (dB/m)	55	249.1 ± 52.7	254	225.0/274.5	94	273.1 ± 61.1	267.0	230.3/320.5	
**Panel b**	**T1D**				**T2D**				
	**N**	**Mean ± SD**	**Median**	**LQ/UQ**	**N**	**Mean ± SD**	**Median**	**LQ/UQ**	
CKD									
Creatinine (mg/dL)	83	0.8 ± 0.18	0.77	0.7/0.9	282	0.9 ± 0.5	0.8	0.7/0.9	
eGFR (CKD-EPI, ml/min/1.73m^2^)	83	100.37 ± 19.84	98.6	90.2/113.7	282	88.4 ± 18.8	91.9	77.6/100.8	
uACR (mg/g)	83	52.51 ± 325.98	6.72	4.5/14.9	280	139.0 ± 734.4	11.3	5.2/27.4	
DSPN^1^									
NDS	81	2.0 ± 2.7	1.0	0.0/2.0	269	3.3 ± 2.3	2.0	1.0/6.0	
NSS	83	2.0 ± 2.9	0.0	0.0/5.0	276	3.9 ± 3.7	5.0	0.0/7.0	
NCV sural nerve (m/s)	77	40.4 ± 15.6	47.0	38.0/50.0	232	35.0 ± 17.8	42.0	11.7/48.0	
SNAP sural nerve (µV)	77	7.9 ± 6.4	7.3	1.8/12.0	232	4.4 ± 4.3	3.1	3.1/8.4	
NCV peroneal nerve (m/s)	75	41.7 ± 4.8	42.0	39.0/45.0	246	41.2 ± 5.5	41.8	38.0/45.0	
CNAP peroneal nerve (mV)	75	6.6 ± 3.3	6.6	4.4/9.2	246	5.8 ± 3.5	5.8	3.0/7.9	
DML peroneal nerve (ms)	75	4.4 ± 0.9	4.2	3.8/4.7	246	4.3 ± 0.9	4.1	3.7/4.6	
NCV tibial nerve (m/s)	62	43.6 ± 4.5	44.0	40.9/46.4	192	40.5 ± 5.2	40.9	37.0/44.0	
CNAP tibial nerve (mV)	62	17.0 ± 7.6	17.5	12.6/22.1	191	11.7 ± 6.9	11.4	6.5/16.3	
DML tibial nerve (ms)	62	3.9 ± 0.8	3.9	3.42/4.3	191	4.1 ± 1.5	3.9	3.5/4.3	
CAN									
E/I-quotient	79	1.22 ± 0.15	1.2	1.1/1.3	261	1.1 ± 0.2	1.1	1.1/1.2	
30/15-quotient	79	1.25 ± 0.24	1.17	1.1/1.5	259	1.2 ± 0.2	1.11	1.0/1.2	
Orthostasis delta systolic	65	−0.2 ± 12.9	1.5	−4.3/5.2	236	0.2 ± 12.0	−1.0	−5.6/5.0	
Orthostasis delta diastolic	66	−3.17 ± 7.14	−2.5	−6.1/0.0	236	−0.9 ± 8.2	−1.5	−4.5/2.4	
Diabetes related RLD									
Vital capacity (%)	78	98.2 ± 15.7	97.5	89.0/106.8	273	99.6 ± 15.5	98.0	90.0/109.0	
Tiffenau-Index (%)	79	96.2 ± 10.4	97.0	90.0/103.0	274	98.6 ± 9.9	100.0	92.0/105.0	
TLC body plethysmography (%)	78	101.4 ± 16.8	100.0	92.0/110.0	272	99.9 ± 15.1	100.0	89.8/109.0	
Diffusing capacity for CO2 (%)	77	84.8 ± 15.8	84.0	74.0/95.0	270	86.0 ± 17.7	85.0	74.0/98.0	
6-min walk-test (m)	60	592.4 ± 77.2	598.0	551.5/636.0	203	525.1 ± 83.8	537.9	471.5/577.6	
MASLD/Liver fibrosis									
Liver stiffness (kPa)	74	4.8 ± 1.5	4.5	3.9/5.6	244	6.6 ± 4.2	5.6	4.4/7.7	
CAP (dB/m)	55	210.4 ± 61.7	205.0	176.0/249.5	148	308.7 ± 60.2	312.5	264.8/356.8	

Number (N) of individuals with normal glucose tolerance (NGT), prediabetes (PRED), type 1 diabetes (T1D) and type 2 diabetes (T2D) for the respective parameter, mean with standard deviation (SD) and median with lower (LQ) and upper quartile (UQ). ^1^ all parameters for NCV stated for the right side.

Abbreviations: CKD: chronic kidney disease, DSPN: diabetic sensorimotor peripheral neuropathy, CAN: cardiovascular autonomic neuropathy, RLD: restrictive lung disease, MASLD: metabolic dysfunction-associated steatotic liver disease, eGFR (CKD-EPI): estimated glomerular filtration rate according to chronic kidney disease epidemiology collaboration, uACR: urinary albumin-to-creatinine ratio, NDS: neuropathy disability score, NSS: neuropathy symptom score, NCV: nerve conduction velocity, SNAP: sensory nerve action potential, CNAP: compound nerve action potential, DML: distal motor latency, E/I-quotient: exhalation/inhalation quotient, TLC: total lung capacity, CAP: controlled attenuation parameter.

### Summary of the results obtained so Far

By mid-2024, based on the HEIST-DiC study 44 articles were published in peer-reviewed journals, reflecting its ongoing contributions to the understanding of diabetes-associated complications. The distribution of these publications across the years is as follows: 1 article in 2017^90^, 4 articles in 2018^49,91–93^, 3 articles in 2019^94–96^, 5 articles in 2020^97–101^, 6 articles in 2021^50,57,102–105^, 7 articles in 2022^12,33,46,106–109^, 7 articles in 2023^38,44,45,58,110–112^, and 11 articles by mid-2024^13,39–43,52,53,113–115^.

### Assessment of classical diabetes-associated complications

- Using MRN, our previous studies have successfully identified fascicle lesions in the sciatic nerve trunk of individuals with T2D and DSPN^116,117^. These lesions were characterized by hyperintense mono- or multifocal patterns, predominantly located at the thigh level. In the HEIST-DiC cohort, we further established that the nerve lesion loads observed through MRN correlated with the severity of clinical symptoms associated with DSPN, as well as with impaired nerve conduction and sensory loss^97^ and with HbA1c^118^. Specifically, MRN-derived parameters, such as fractional anisotropy, which indicate nerve fiber integrity, demonstrated strong correlations with increased NDS and decreased nerve conduction velocity, independent of factors like age, sex, BMI, and HbA1c^58^. These findings suggest that MRN can serve as a valuable non-invasive diagnostic tool for evaluating nerve function in DSPN and DM. This method offers significant advantages over traditional assessment methods, such as standardized questionnaires and clinical examinations, which are often reliant on subjective evaluations and individuals’ cooperation.
- Another significant finding from our research is the demonstration that QST can effectively identify early sensory deficits in individuals with and without T2D. These deficits were found to be associated with markers of insulin resistance, metabolic syndrome, and glycation end-products^44^. Based on QST data analysis, we confirmed four sensory phenotypes: healthy, thermal hyperalgesia, mechanical hyperalgesia, and sensory loss. Longitudinal analysis of these QST-based sensory phenotypes, provided valuable insights into the natural progression of DSPN, with the sensory loss phenotype being the most strongly correlated with DSPN^43^. Additionally, MRN of T2D individuals who exhibited the most severe sensory phenotypes (mechanical hyperalgesia and sensory loss) revealed diminished structural integrity of the sciatic nerve. This structural decline appears to precede the sensory loss observed in peripheral nerves, highlighting the potential of MRN as a tool for early detection and monitoring of nerve integrity in the context of diabetes-associated complications^41^.
- Regarding CKD, our longitudinal analysis indicated a progression of CKD over a 4-year period. Specifically, we observed that 6% of individuals with PRED experienced CKD progression, while 12% of individuals with T2D without previously known CKD also showed similar progression. Notably, 46% of individuals with T2D who had existing CKD experienced a worsening of their condition^38^. Furthermore, we found that albuminuria could be temporarily improved through dietary interventions in individuals with T2D and known CKD. This improvement in albuminuria was associated with significant changes in parameters related to fatty acid oxidation and was not sustained after the end of dietary intervention. These findings indicate that dietary management could play a role in the treatment of renal complications in DM^33^.
- Regarding MASLD, a 4-year longitudinal analysis showed that 24% of individuals with PRED experienced progression, indicated by worsening of liver stiffness. This was also observed in 19% of participants with T2D without increased liver stiffness at baseline and 15% of those with T2D and increased liver stiffness at baseline. Interestingly, 36% of the participants without known diabetes-associated complications also exhibited increased liver stiffness^38^.

### Assessment of non-classical diabetes-associated complications

- Our study identified a significant increase in breathlessness and a notable prevalence of RLD among individuals with long-term T2D (27%). In comparison, 21% of individuals with recent-onset T2D and 9% of individuals with PRED showed similar patterns of lung disease^49^. To confirm the presence of interstitial lung disease, we utilized multidetector computed tomography, and histological analysis revealed evidence of fibrosis in the lungs of individuals with T2D^49^. These findings align with previous studies that have linked a decline in pulmonary function with T2D^119,120^. Our results underscore the importance of recognizing diabetic RLD as a critical component in the standard care for managing diabetes-associated complications^50^.
- We showed that that DSPN does not solely affect the lower limbs; it also significantly impacts the upper limbs, making them susceptible to diabetes-induced damage. The sensory phenotype observed in individuals with upper limb neuropathy closely resembles that of lower limb neuropathy, primarily characterized by loss of sensory function^107^.
- We found that diabetes-related distress was significantly associated with lower glycemic control, higher insulin resistance, and longer DM duration in individuals with T2D^13^. Additionally, we demonstrated an impairment in the autonomic nervous system’s response to stress, which is partially reflected in the psychological stress response^12^. Importantly, we found a notable association between moderate to severe childhood neglect and an intensified psychological stress response in individuals with T2D. This connection highlights the interplay between psychological factors, physiological stress responses, and childhood experiences in the context of DM management, suggesting the need for comprehensive approaches addressing both emotional and physical health in T2D individuals^105^.

### Identification of biomarkers for diabetes-associated complications

- In our study, we identified circulating mRNA levels of myelin protein zero as a novel potential biomarker associated with DSPN, which together with the already reported biomarker for DSPN, neurofilament light chain protein, were found changed in our cohort; specifically, decreased levels of myelin protein zero were predictive of hypoalgesia, while increased levels of neurofilament light chain were linked to a hyperalgesia phenotype^57^. Furthermore, neurofilament light chain protein levels correlated with sensorimotor deficits in both the upper and lower limbs in individuals with T2D^58^. These findings suggest that these biomarkers could be valuable in assessing neuropathic pain and sensory function in diabetic individuals.
- We demonstrated that the bioelectrical phase angle derived from BIA can serve as a straightforward tool for assessing cardiovascular risk and detecting DSPN in individuals with and without DM^52,53^. This finding highlights the phase angle’s potential utility in clinical settings for early identification of individuals at risk for cardiovascular complications and neuropathy, aiding in timely interventions and management strategies.

### Novel cellular mechanisms linked to diabetes-associated complications and precise interventions

The traditional glucose-centric approach to diabetes management, which focused on lowering glycemia, has been increasingly challenged, leading to a shift toward a holistic, patient-centered approach aimed at reducing the risks of diabetes-related complications^121^. This framework acknowledges the complexity of diabetes as a chronic disease and emphasizes the importance of a pathogenesis-centric strategy, targeting the underlying pathophysiological mechanisms that both cause and complicate diabetes and its associated complications^122^.

- Cross-sectional analysis of individuals with PRED and T2D showed a strong association between the presence of CKD, RLD phenotype, and increased liver stiffness with elevated markers for DNA damage, senescence, and senescence-associated secretory phenotype (SASP)^38^. Furthermore, longitudinal analysis over four years revealed that the progression of CKD was significantly predicted by these markers of DNA damage, senescence, and SASP. In contrast, the progression of RLD was primarily associated with increased DNA damage and elevated levels of interleukin-6 (IL-6)^38^. These findings suggest that cellular aging plays a critical role in the complications observed in individuals with diabetes.
- In the HEIST-DiC cohort, we successfully validated a novel p21-dependent mechanism of tubular senescence previously identified in animal models, which contributes to hyperglycemic memory in the context of CKD and T2D^109,115^. We observed that tubular and urinary p21-levels from individuals with T2D were significantly associated with the severity of CKD. Notably, these p21 levels remained elevated even when blood glucose levels improved through treatment with SGLT-2 inhibitors or dietary interventions^33,109,115^. This persistence of p21 elevation, despite better glycemic control, underscores the potential for tubular senescence to play a pivotal role in the progression of CKD.
- We discovered elevated hydroxyacetone levels and increased activity of aldo-keto-reductase in red blood cells of T2D individuals, as markers associated with compensatory mechanisms for methylglyoxal detoxification. These markers may serve as useful indicators to distinguish between T2D individuals with and without complications. Specifically, individuals with T2D who do not exhibit complications appear to retain protective alternative detoxification pathways for methylglyoxal, which likely contributes to their better health profile. Conversely, those with T2D complications show a loss of these protective mechanisms, suggesting that impaired detoxification processes may contribute to the progression of diabetes-associated complications^91^.
- In a subgroup of individuals with T2D and CKD we conducted a randomized-controlled trial focusing on the effects of a diet intervention involving periodic fasting over 6 months. This study aimed to evaluate outcomes related to diabetes-associated complications, particularly the impact on microalbuminuria and somatosensory nerve function^33,112^. Our findings indicated that improvement of microalbuminuria under periodic fasting was linked to specific changes in acylcarnitine profile^33^, and had no effect on somatosensory nerve function^112^. Additionally, the effectiveness of periodic fasting on weight loss and maintenance appeared to be influenced by a genetic polymorphism of p53^113^. In a following *proof-of-concept* study we demonstrated that glucose intake during refeeding after periodic fasting led to an increased oxidative stress response in T2D individuals with complications^114^. T2D individuals without complications seemed to be unaffected by the glucose-induced changes, whereas individuals with NGT experienced enhanced cellular resistance to oxidative stress^114^. These results suggest that while periodic fasting may offer benefits for managing microalbuminuria in T2D individuals with CKD, the oxidative stress response post-refeeding could vary significantly based on the presence of diabetes-associated complications.

Taken together, our findings are significant as they shift the focus from the traditional glucose-centered hypothesis to a broader understanding of the various mechanisms influencing the development and progression of diabetes-associated complications. By identifying these novel mechanisms, we open avenues for designing targeted interventions aimed at preventing, improving, or even achieving remission of these complications.

### What are the main strengths and limitations?

The HEIST-DiC cohort includes individuals across the full spectrum of glucose metabolism, offering a unique opportunity to address critical gaps in diabetes research, such as refining clustering methods, assessing subtype stability, and identifying biomarkers and high-risk individuals. By stratifying participants based on pathophysiological mechanisms, the cohort bridges epidemiological studies and clinical research. This approach will validate existing methods and drive new frameworks, supporting tailored therapeutic strategies and advancing precision medicine in diabetes care. The main strengths of this cohort include: (i) the inclusion of individuals exhibiting a broad diversity in metabolic and clinical stages, providing in this way a comprehensive representation of clinical trajectories of PRED, T1D and T2D; (ii) extensive metabolic and clinical phenotyping for classical and non-classical diabetes-associated complications; and (iii) an extended follow-up period of 11 years. Consequently, the HEIST-DiC cohort presents the opportunity to examine the natural course of DM while simultaneously investigating the onset and progression of diabetes-associated complications and differentiating between fast and slow progressors.

The comprehensive metabolic and clinical phenotyping of diabetes-associated complications has enabled us to implement novel methods by flexibly adapting the study protocol to current findings and refining the focus based on these insights. For instance, in December 2016, we incorporated the 6-minute walk test to assess emerging lung complications related to diabetes. Hand nerve conduction velocity was added in February 2021 in response to emerging evidence of impaired nerve function in the upper extremities. In June 2023, transcutaneous electrical nerve fiber stimulation was introduced to evaluate small nerve fibers (Table 2). The method we employ selectively assesses C-nociceptor excitability through slow, long-duration pulses at low frequency^123,124^. Alternative novel techniques utilizing short, high-frequency rectangular pulses, primarily assessing A-fiber function, should be considered in future protocol adaptations to allow for more comprehensive assessment of peripheral sensory function^125^.

This thorough characterization allows to design precise intervention studies aimed at prevention, improvement or ideally, remission of diabetes-associated complications.

Another notable strength is provision and explanation of detailed results to study participants, which they can share with their healthcare providers. This practice keeps participants informed about their health status and enhances their compliance, further supported by regular educational events organized by the study team on DM awareness and information. On the other hand, the communication of the detailed results to the healthcare providers may prompt adjustments in treatment strategy. Additionally, the annual study visits may increase personal motivation and adherence to therapeutic recommendations, thereby incorporating intervention elements compared to study participants.

HEIST-DiC provides more intensive assessments with annual clinical visits, compared to the German Diabetes Study (GDS), which conducts clinical evaluations every five years^71^. Unlike the Diabetes Prospective Follow-up Registry (DPV)—a registry collecting demographic, clinical, and treatment data for T1D and T2D since 1995—HEIST-DiC offers detailed insights into diabetes-related complications using advanced methods available only in specialized university research centers^126^. While the Verona Diabetes Study and the Diabetes Education and Self-Management for Ongoing and Newly Diagnosed (DESMOND) program focus on mortality and self-management, their follow-up periods are shorter, at five years and three years respectively^127,128^. While the current follow-up period is set at 11 years, future extensions will be implemented in response to emerging scientific questions or the integration of novel methodologies.

While predicting long-term disease trajectories in individuals aged 85 and older may have limited applicability, choosing a high upper age limit ensures inclusivity and better reflects the real-world population seen in clinical practice. Older adults are frequently underrepresented in clinical research, despite bearing a significant burden of chronic diseases such as T2D. Including them allows to capture age-related variability and potentially identify important trends within this population. Furthermore, with regard to diabetes subtypes, individuals with mild age-related diabetes are typically older and present with milder metabolic disturbances compared to other subtypes^14,37^. This approach opens the possibility of identifying a subgroup of individuals with diabetes who are less susceptible to complications and may achieve healthy aging despite the disease. Establishing and further characterizing this subgroup using advanced analytical tools represents an important goal for future research.

As the majority of participants are recruited from our outpatient university clinic, a potential source of bias may arise from the inclusion of individuals with a longer duration of diabetes and the presence of pre-existing diabetes-associated complications. However, these individuals provide valuable insights into the development of DM and the associated complications, without the beforementioned intervention elements inflicted by the monitoring during a study.

One limitation of the HEIST-DiC study is the insufficient representation of diverse ethnicities, as it exclusively includes individuals residing in Germany and does not record ethnicity data. Additionally, our cohort comprises more female participants in the NGT group, which is currently addressed by strategic recruitment of more male participants.

Given that the HEIST-DiC study is a hypothesis-generating cohort study, the initially conducted sample size calculations are based on a logistic regression model for a binary outcome with a single binary or continuous covariate. Typically, cohort studies incorporate additional co-variables that may act as confounders, which should be integrated into the regression model. This oversight reduces the statistical power of the analysis, necessitating adjustments to the initially calculated sample sizes using the variance inflation factor and a factor accounting for expected dropout rates^129^. As Heist-DiC adapts its study protocol in response to emerging findings, the inclusion of new target groups and outcomes will be continuously considered throughout the course of the study. Consequently, the final sample sizes may need to be larger than originally calculated. Heist-DiC was intentionally designed as an open-end hypothesis-generating cohort study, without a fixed participant target or end date, to maintain flexibility. This design also supports the potential inclusion of new target groups, correction of imbalances in existing groups, and replacement of drop-outs—ensuring an active cohort and supporting the study’s function as a recruitment platform for intervention studies.

Study investigators are not required to evaluate clinical findings beyond the parameters of the research question, an approach that is approved by the ethics committee and is clearly explained to participants prior to their inclusion in the study. Assessment of preclinical manifestation of diabetes-associated complications or other comorbidities may not result in immediate changes in clinical management. In case any relevant incidental abnormal finding is detected during magnetic resonance imaging, ultrasound or in blood chemistry, participants are informed and, if requested, the information is reported to their healthcare providers for further evaluation.

## Supplementary Information

Below is the link to the electronic supplementary material.

Supplementary Material 1

## Data Availability

The datasets generated and analyzed during the current study are not publicly available due to national data protection laws but are available from the corresponding author on reasonable request. Researches may apply for data and/or biological samples by contacting the study coordinators via email (Stoffwechsel.Studien@med.uni-heidelberg.de). The steering committee of the study will evaluate the request. After approval, the requesting researcher and the principal investigator of HEIST-DiC sign a contract on data transfer and transmission of results.

## Abbreviations

ADA: American Diabetes Association
Adipo-IR: Adipose tissue insulin resistance index
ALAT: Alanine aminotransferase
ASAT: Aspartate aminotransferase
BIA: Bioelectrical impedance analysis
BP: Blood pressure
CAN: Cardiovascular autonomic neuropathy
CKD: Chronic kidney disease
CKD-EPI: Chronic Kidney Disease Epidemiology Collaboration
CNAP: Compound nerve action potential
Diast: Diastolic
DL_CO_: Diffusing capacity of the lung for carbon monoxide
DM: Diabetes mellitus
DML: Distal motor latency
DR: Diabetic retinopathy
DSPN: Distal sensorimotor polyneuropathy
eGFR: Estimated glomerular filtration rate
E/I: Exhalation / inhalation
ESC: European Society of Cardiology
FFA: Free fatty acids
FLI: Fatty-liver-index
FIB-4: Fibrosis-4 index
GGT: Gamma glutamyl transferase
GLP-1: Glucagon-like peptide 1
hsCRP: High-sensitivity C-reactive protein
hsTNT: High-sensitivity troponin T
HbA1c: Glycated hemoglobin
HDL: High-density lipoprotein
HIV: Human immunodeficiency virus
HRV: Heart rate variability
ins sens: Insulin sensitivity
KDIGO: Kidney Disease: Improving Global Outcomes
LADA: Latent autoimmune diabetes of the adult
LDL: Low-density lipoprotein
Lp(a): Lipoprotein (a)
MASLD: Metabolic dysfunction-associated steatotic liver disease
MASLDS: Metabolic dysfunction-associated steatotic liver disease score
MODY: Maturity onset diabetes of the young
MNSI: Michigan neuropathy screening instrument
MRN: High-resolution magnetic resonance neurography
NCV: Nerve conduction velocity
NDS: Neuropathy disability score
NGT: Normal glucose tolerance
NSS: Neuropathy symptom score
NTproBNP: N-terminal pro B-type natriuretic peptide
NYHA: New York Heart Association
oGTT: Oral glucose tolerance test
PPAR: Peroxisome proliferator-activated receptor
PHQ: Patient health questionnaire
PRED: Prediabetes
QST: Quantitative sensory testing
RLD: Restrictive lung disease
SAS: Symptom assessment score
SASP: Senescence-associated secretory phenotype
SF-12: 12-item short-form health survey
SGLT-2: Sodium-glucose cotransporter-2
SIRD: Severe insulin-resistant diabetes
SIDD: Severe insulin-deficient diabetes
SNAP: Sensory nerve action potential
Syst: Systolic
T1D: Type 1 diabetes
T2D: Type 2 diabetes
TLC: Total lung capacity
uACR: Urinary albumin-to-creatinine ratio
