# Supplementary material for: Cohort profile of the Heidelberg study on diabetes and complications HEIST-DiC
Source: Sci Rep. 2025 Aug 12;15:29580. doi: 10.1038/s41598-025-15343-8 (PMC12343885; doi:10.1038/s41598-025-15343-8)
Supplement: Supplementary file 1 — Supplementary Material 1 [file 41598_2025_15343_MOESM1_ESM.docx]

**Supplementary Material**

**Methods**

1. **Medical history and anthropometric measurements**

Previous illnesses, allergies, medical history of diabetes (time of diagnosis, diagnosed diabetes-associated complications, family history), medication, smoking habits (current amount, duration and time since cessation for former smoking) and alcohol consumption (amounts, regularity, kind of beverage) are assessed by physicians.

Measurement of body height with a stadiometer and weight using a calibrated scale (MPD 250K100M, KERN & SOHN GmbH, Balingen-Frommern, Germany) is performed without shoes, with empty pockets, and fully dressed. Weight is adjusted by subtracting 0.5 kg to adjust for clothes. Waist circumference is taken midway between lower costal arch and the superior anterior iliac spine and hip circumference at the widest point over the greater trochanters using a non-elastic tape.

1. **Chronic kidney disease**
   1. **Kidney Ultrasound**

Kidney ultrasound is performed to measure kidney size, cortical thickness, as well as to assess morphology of the kidney; doppler ultrasound of the renal arteries is performed to measure the resistive index (3.5 Mhz 4C RS standard transducer, Versana Premier^TM^ R2 VS, GE Medical Systems SCS, France; before April 2021: SonoAce 8000SE, Samsung Medison Co., Ltd., Seoul, Republic of Korea).

1. **Distal sensorimotor polyneuropathy**
   1. **NDS/NSS/MNSI**

Anamnestic assessment of neuropathic complaints is performed with neuropathy symptom score (NSS) and michigan neuropathy screening instrument (MNSI) questionnaires, and clinical assessment through clinical neurological examination according to the neuropathy disability score (NDS) and MNSI questionnaires (1; 2).

- 1. **Purdue pegboard test**

Individuals are encouraged to place as many items on the Purdue Pegboard^TM^ (Model 32020A, Lafayette Instrument Company, USA) as possible in the given time. In three 30-second rounds a pin is placed with 1) the right hand, 2) the left hand, 3) both hands. Afterwards there is 1 min available to place a pin with the right hand and stack a disc, a cylinder and a second disc while alternating hands. Number of placed items for each round is recorded (3).

- 1. **Peripheral nerve electrophysiological conduction**

As part of the electrophysiological assessment of the lower extremities, minimal electrical currents are applied via adhesive skin electrodes using the Viking IV Electromyography System (Viasys Healthcare VikingQuest®, Viasys Healthcare GmbH, Höchberg). For the sensory assessment of the sural nerve, nerve conduction velocity and sensory nerve action potential are measured. In the motoric examination of the peroneal and tibial nerve, nerve conduction velocity, compound muscle action potential and distal motor latency are recorded (4). For the hand, sensory and motor component on the median and ulnar nerves were measured (5). Skin temperature was at least 32°C throughout the examination.

- 1. **Transcutaneous electrical nerve fiber stimulation**

Electrical stimuli were delivered through bipolar platinum electrodes (0.4 mm in diameter, spaced 2 mm apart). A constant current stimulator (Digitimer DS5, Welwyn Garden City, UK), connected to a digital-to-analogue converter (DAQ NI USB-6221, National Instruments, Austin, TX, USA), generated the sinusoidal and half-wave pulses, all controlled by specialized software (Dapsys8 software, © Brian Turnquist, Bethel University, USA). For each stimulus patients rated the corresponding pain intensity on a numeric rating scale (NRS) with the endpoints 0 (no pain) and 10 (maximum pain that can be imagined).

Electrical stimulation protocol: single half-sine wave stimuli were administered with set durations, while intensity varied randomly between 0.2 and 1 mA. This was followed by sinusoidal stimuli at 4 Hz, with incremental increases in intensity until participants detected the stimuli, establishing their detection threshold. The intensity was then raised further until participants rated the pain at a level of 2-3 on the NRS, marking their pain threshold. In the next phase, additional sinusoidal stimuli were applied at various intensities, with participants rating perceived pain. Finally, continuous supra-threshold sinusoidal stimulation was delivered, with pain intensity recorded at specific intervals (5, 15, 30, 45, and 60 seconds). Each assessment, including both sinusoidal and half-sine wave stimuli, was repeated twice per intensity level, and the mean values were calculated for analysis (6; 7).

- 1. **Quantitative sensory testing**

Quantitative sensory testing (QST) is carried out according to the protocol of the German Neuropathic Pain Research Network (DFNS) (8). The tests include thermal detection thresholds for the perception of cold, heat and paradoxical heat sensations and thermal pain thresholds for cold and hot stimuli, which are all measured with a Thermal Sensory Analyzer (TSA-II, Medoc Ltd, Israel). Mechanical detection thresholds for light touch, vibration and mechanical pain sensitivity (threshold determination for blunt pressure and pinprick stimuli, stimulus-response curve for pinprick stimuli, dynamic allodynia, pain summation for repeated pinprick stimulation) are determined with a set of von Frey-filaments (0.25, 0.5, 1, 2, 4, 8, 16, 32, 64, 128, 256 und 512 mN; OptiHair_2_, MRC Systems GmbH, Heidelberg, Germany), pinprick punctate probes (8, 16, 32, 64, 128, 256 und 512 mN; PinPrick, MRC Systems GmbH, Heidelberg, Germany), cotton pad, 15 cm Q-tip, brush (SENSELab Brush-05, Somedic SenseLab AB, Sösdala, Sverige), tuning fork (64 Hz, 8/8 Skala, Aesculap B. Braun SE, Melsungen, Germany ) and a mechanical force gage (Force Dial^TM^ FDK/FDN, Wagner Instruments, Greenwich, USA). All 13 domains are measured on the right foot and for control on a contralateral area on the hand.

- 1. **High-resolution magnetic resonance neurography**

Magnetic resonance imaging (MRI) of the right thigh musculature is performed in a 3.0 Tesla MR-scanner (Magnetom TRIO, Siemens Healthineers, Erlangen, Germany). Individuals are examined in supine position with their feet first. Contrast agent to measure nerve perfusion and permeability is administered (Dotarem^®^, Guerbet, France, 0.1 mmol/kg, flow rate 3.5ml/s). A 15-channel transmit-receive extremity coil is used to apply two sequences (Axial high-resolution T2-weighted turbo spin echo (TSE) 2D sequence with spectral fat suppression; Axial T1-weighted volume interpolated breath-hold examination (VIBE) sequence). The sequences are centred to the sciatic nerve bifurcation at distal thigh level. Images of the T2-weighted sequence are manually segmented with ImageJ and co-registered to the T1 VIBE sequence with affine transformations using custom-written code in Matlab (MathWorks, Natick, MA, R2020b). The extended Tofts model is used to asses permeability of the nerve using the constant of permeability, extravascular extracellular volume fraction and plasma volume fraction (9; 10).

1. **Cardiovascular autonomic neuropathy**
   1. **Heart Rate Variability**

Cardiovascular autonomic nerve function is assessed by measuring heart rate variability during spontaneous breathing, deep breathing (expiration/inspiration ratio) and standing up (max/min 30/15 ratio, orthostatic hypotension) (SUESS SUEmpathy® 100, SUESS Medizin-Technik GmbH, Aue, Germany) (11).

- 1. **SAS**

Anamnestic assessment of cardiovascular autonomic neuropathy is performed with the symptom assessment score (SAS) questionnaire (12).

1. **Diabetic retinopathy**
   1. **Fundoscopy**

Central 1-field fundus photography with a 45° or 37° field (Non-Mydriatic Auto Fundus Camera, NIDEK AFC-230®, NIDEK CO., LTD., Maehama, Japan equipped with Canon EOS 5D Mark II, Canon Germany GmbH, Krefeld, Germany) is used to capture digital fundus images. Application of eye drops is not required.

1. **Diabetic pneumopathy**
   1. **Clinical assessment**

Clinical assessment of the lungs includes auscultation of the lungs using a high-quality stethoscope in upright seated position to facilitate optimal lung expansion.

- 1. **6-minute walk test**

Individuals are encouraged to walk as far as possible in 6 minutes. The covered distance is recorded by a walking wheel (Velometer 305, Weiss Messwerkzeuge GmbH, Erbendorf, Germany). Before and after walking, dyspnoea is assessed using the BORG CR-10 scale (13) and oxygen saturation is measured using a finger clip (Pulox® PO-250, Novodion GmbH, Cologne, Germany).

- 1. **Pulmonary function tests**

Spirometry, body plethysmography and carbon monoxide-based diffusion capacity are recorded using a body plethysmograph (Ganshorn PowerCube® Body+ and Diffusion+, Ganshorn Medizin Electronic GmbH, Niederlauer, Germany) according to the manufacturers protocol.

1. **Liver steatosis and fibrosis**
   1. **Transient elastography**

Measurement of liver stiffness and controlled attenuation parameter are performed using elastography (Echosens FibroScan^®^, Paris, Echosens™, Frankreich) with the probe appropriate for the individual’s BMI (M: BMI <30 kg/m^2^, XL: BMI >=30 kg/m^2^).

- 1. **Hepatic sonography**

Liver ultrasound is performed to measure liver size and to assess for steatosis hepatis (3.5 Mhz 4C RS standard transducer, Versana PremierTM R2 VS, GE Medical Systems SCS, France; before April 2021: SonoAce 8000SE, Samsung Medison Co., Ltd., Seoul, Republic of Korea).

1. **Vascular assessment**
   1. **Clinical examination**

Palpation of pulses on both legs is performed for clinical examination of peripheral circulation: femoral artery, popliteal artery, dorsal artery of the foot, posterior tibial artery.

- 1. **24h-Blood pressure measurement**

Continuous blood pressure is measured on the upper arm 2-3 cm above the crook of the arm for 24 hours (boso TM-2430, Bosch + Sohn GmbH u. Co. KG, Jungingen, Germany).

- 1. **Ankle-brachial index**

Blood pressure is measured on both arms and legs using the Riva-Rocci method (BOSO ABI System 100, Bosch + Sohn GmbH u. Co. KG, Jungingen, Germany). Arm cuffs are placed on both upper arms 2-3 cm above the crook of the arm and on both legs 1-2 cm above the ankle.

- 1. **Intima media thickness and carotid ultrasound**

Using ultrasound (3.5 Mhz L6-12 RS standard transducer, Versana Premier^TM^ R2 VS, GE Medical Systems SCS, France; before April 2021: SonoAce 8000SE, Samsung Medison Co., Ltd., Seoul, Republic of Korea), the flow profiles and flow velocities of the common, internal and external carotid arteries are examined on both sides for the presence of plaques or stenoses and intima-media thickness is measured. The degree of stenosis is categorized according to the current NASCET criteria.

- 1. **12-lead resting electrocardiogram**

The patient rests in a comfortable position, usually lying supine. The skin areas where electrodes will be placed are cleaned with alcohol wipes to ensure good conductivity. Four electrodes are placed on the limbs: right arm, left arm, right leg, left leg. Six electrodes are placed on the chest: V1: 4th intercostal space at the right sternal border, V2: 4th intercostal space at the left sternal border, V3: Midway between V2 and V4, V4: 5th intercostal space at the midclavicular line, V5: 5th intercostal space at the anterior axillary line, V6: 5th intercostal space at the midaxillary line (Mortara Instrument ELI250, Mortara Instrument GmbH, Essen, Germany).

1. **Muscle strength and body composition**
   1. **Hand grip strength**

Hand grip is measured by pressing a hydraulic hand dynamometer (200 lb., Baseline STD hydraulic hand dynamometer, Fabrication Enterprises Inc, Elmsford, USA).

- 1. **Bioelectrical impedance analysis**

Electrodes are attached to the individuals’ skin, on the back of both hands and feet according to the manufacturer’s manual, and body tissue composition and bioelectrical impedance of the body are measured by application of small electrical currents (Biacorpus® RX 4004M, MEDI CAL HealthCare GmbH, Karlsruhe, Germany).

- 1. **AGE measurement**

Non-invasive fluorescence-based measurement of advanced glycation end products in the skin (AGE Reader SU, Diagnostics Technologies B.V., Groningen, Netherlands) by placing the inside of the forearm on the sensor field.

1. **Metabolic testing**

All tests are performed after over-night fasting.

- 1. **Oral glucose tolerance test (oGTT)**

A venous access (BD Venflon^TM^ Pro Safety 20G or 22G, BD, Franklin Lakes, USA) is inserted into an antecubital vein. After a fasting blood draw, the individual drinks 75 g of glucose in a freshly prepared standardized 300 ml solution (GLUKO 75 Pulver, MEDICALFOX GmbH, Hamburg, Germany). Glucose, insulin and C-peptide are measured at 0, 60, 90 and 120 min.

- 1. **Intravenous glucose tolerance test (IVGTT)**

After baseline blood sampling 1 ml/kg body weight of glucose 30% (prepared with glucose 40%, SERAG-WIESSNER GmbH & Co. KG, Naila, Germany) is administered as a bolus through an intravenous access on the right arm (BD Venflon^TM^ Pro Safety 20G or 22G, BD, Franklin Lakes, USA) and the blood glucose is monitored every 2 minutes until minute 10 and then every 10 minutes until minute 60. Insulin and C-peptide are measured at baseline and minutes 4, 10 and 60. First phase C-peptide secretion until minute 10, second phase C-peptide secretion until minute 60 and total C-peptide secretion are calculated as incremental area under the curve.

- 1. **Euglycemic hyperinsulinemic clamp**

The hyperinsulinemic-euglycemic clamp test is the gold-standard measure for whole-body insulin sensitivity (14). Following the IVGTT, the clamp test starts with an insulin bolus (10 mU insulin / kg body weight *min for 10 min) and continues with a steady infusion of short-acting human insulin (Insuman® Rapid sanofi-aventis Frankfurt am Main Germany) (1.5 mU/kg body weight*min for at least 3 h) via an intravenous access on the right arm (BD Venflon^TM^ Pro Safety 20G or 22G, BD, Franklin Lakes, USA) (15). Blood glucose concentration is determined manually every 5 min and adjusted by regulation of an intravenous infusion of 20% glucose (G-20%, B. Braun Melsungen AG, Melsungen, Germany) in the right arm maintaining a target value of 90 mg/dl. Space-corrected mean whole-body glucose disposal (M-value) is calculated from the glucose infusion rate during the last 30 min of the clamp.

- 1. **Hepatic insulin sensitivity**

Hepatic insulin sensitivity is determined by means of continuous deuterated glucose tracer infusion (D-[6,6-^2^H_2_]-glucose) (Cambridge Isotope Lab., Andover, Mass., USA; 99% ^2^H enriched) as insulin-suppressed endogenous glucose production (16). Infusion of deuterated glucose (D-[6,6-^2^H_2_]-glucose) is started two hours before the start of the IVGTT by bolus administration over 10 minutes followed by continuous infusion of D-[6,6-^2^H_2_]-glucose aiming at enrichment of 2%. All other infusion solutions used for IVGTT and clamp must also be enriched by 2% of (D-[6,6-^2^H_2_]-glucose). Determination of atom percent enrichment (APE) of 2H and calculation of endogenous glucose production are done as described before (17). The analyses are performed on a Hewlett-Packard 6890 gas chromatograph equipped with a 25-m CPSil5CB capillary column (0.2 mm i. d., 0.12 µm film thickness; Chrompack/Varian, Middelburg, The Netherlands) and interfaced to a Hewlett Packard 5975 mass selective detector.

- 1. **Indirect calorimetry**

Following a resting period of 10 minutes, individuals’ heads are placed under the canopy of the respiratory gas analyzer (Quark RMR, COSMED Deutschland GmbH, Fridolfing, Germany) for 15 min. The measurement of O_2_ uptake (VO_2_) and CO_2_ (VCO_2_) output is performed under resting conditions in the fasting state and in the last half hour of the hyperinsulinemic-euglycemic clamp test to determine total energy expenditure, glucose and lipid oxidation (18). An individual calibration for each individual is conducted before the first test and required for the accurate calculation of the resting energy expenditure. The ICcE-corrected values for the primary parameter VCO_2_ and VO_2_ allow to calculate the corresponding resting energy expenditure (REE) by using the *Weir equation* (REE = (3,941*V_O2_+1.11*V_CO2_)*1.44) (19).

1. **Biobank**
   1. **Blood and urine samples**

Samples are taken from over-night fasted individuals. Blood is taken with a Safety-Multifly®-Needle (Safety-Multifly®-Needle 23G or 21G, SARSTEDT AG & Co. KG, Nümbrecht, Germany). Aliquots of whole blood, plasma, lymphocytes and erythrocytes are obtained from EDTA-blood (S-Monovete® 9 ml K3E, SARSTEDT AG & Co. KG, Nümbrecht, Germany). Aliquots of serum are obtained from gel-serum (S-Monovete® 7.5 ml Z-Gel) and urine from Urin-Monovette® 8.5 ml. Plasma and serum samples are processed and stored within 30 min of blood draw. Additional plasma samples are collected at 120 min of OGTT and at the end of the steady state of the clamp test. All samples are stored at -80 °C until further analysis.

- 1. **Skin biopsy**

A 3 mm punch biopsy (Cutaneous Biopsy Punch, pfm medical GmbH, Köln, Germany) is taken under local anaesthesia (Scandicain 2%, Aspen Germany GmbH, München, Germany). The biopsy site is located 10 cm above the outer left ankle (lateral malleolus). Biopsy is processed with incubations in PFA, sucrose and glycerine solution, embedded in tragant and after initial freezing in liquid nitrogen transferred to -80 °C until further analysis (20).

- 1. **Skeletal muscle biopsy**

The biopsy is taken above the vastus lateralis of the quadriceps muscles under local anaesthesia (Scandicain 2%, Aspen Germany GmbH, München, Germany) using a Bergström needle (biopsy cannula 4.0mmx100mm, Mercian Surgical Supply Co Limited, Bromsgrove, UK) via a small skin incision. Approximately 70-400 mg of tissue is collected and stored immediately in liquid nitrogen before transferal to -80 °C until further analysis. Thereafter the incision is bandaged.

- 1. **Adipose tissue biopsy**

The subcutaneous abdominal (periumbilical) adipose tissue biopsy is performed under local anaesthesia (Scandicain 2%, Aspen Germany GmbH, München, Germany) via a small skin incision using an aspiration needle biopsy (Needle 14Gx4 ¾” 2.0x120mm, Vitrex Medical A/S, Herlev, Denmark). Approximately 0.5-2.0 g of fat tissue are collected and stored in liquid nitrogen before transferal to -80 °C until further analysis. Thereafter the incision is bandaged.

1. **Health-related quality of life and depression**

Health-related quality of life is assessed by the 12-Item Short-form Health Survey and depression by the Patient Health Questionnaire (21; 22).

1. **Clinical chemistry**

Standard parameters of clinical chemistry were measured in the central laboratory of the university hospital. In July 2023 the automated system used for the majority of parameters was changed. Most parameters from Na-fluoride, heparin-plasma, urine and serum were previously measured on Siemens Advia Chemistry XPT or Siemens Advia Centaur XPT and changed to Siemens Atellica Solution CH or Siemens Atellica Solution IM (Siemens Healthineers International AG, Zürich; Switzerland). Measurements in citrate plasma changed from Sysmex CS5100 to Sysmex CN6000 (Sysmex Corporation, Kobe, Japan). Blood count changed from Siemens Advia2120i (Siemens Healthineers International AG, Zürich; Switzerland) to Sysmex XN1000 (Sysmex Corporation, Kobe, Japan). Troponin T changed from Roche Cobas e601 to Roche Cobas e801 pro (Roche Diagnostics, Mannheim, Germany). Urine test stripes and urine sediments changed from Siemens Clinitek Novus (Siemens Healthineers International AG, Zürich; Switzerland) respectively Beckman Coulter iQ200 (Beckman Coulter, Inc., Brea, USA) to Siemens Clinitek Advantus (Siemens Healthineers International AG, Zürich; Switzerland). HbA1c is measured on a Variant II Turbo (Bio-Rad, Munich, Germany). sTFR is measured on a Roche Cobas Integra (Roche Diagnostics, Mannheim, Germany). hsCRP is measured on a Dade Behring BN2 (Siemens Healthineers International AG, Zürich; Switzerland). suPAR is measured with suPARnostics AUTO Flex ELISA (ViroGates A/S, Birkerød, Denmark). Additionally, the calibration for cholinesterase was changed in September 2018, with a median increase of 13%, and standardisation for cystatin C was changed in June 2019, resulting in a median increase of 16%.

**Literature**

1. Young M, Boulton A, MacLeod A, Williams D, Sonksen P. A multicentre study of the prevalence of diabetic peripheral neuropathy in the United Kingdom hospital clinic population. Diabetologia 1993;36:150-154

2. Feldman EL, Stevens M, Thomas P, Brown M, Canal N, Greene D. A practical two-step quantitative clinical and electrophysiological assessment for the diagnosis and staging of diabetic neuropathy. Diabetes care 1994;17:1281-1289

3. Tiffin J, Asher EJ. The Purdue Pegboard: norms and studies of reliability and validity. Journal of applied psychology 1948;32:234

4. Gibbons CH, Freeman R, Veves A. Diabetic neuropathy: a cross-sectional study of the relationships among tests of neurophysiology. Diabetes Care 2010;33:2629-2634

5. Gazioglu S, Boz C, Cakmak VA. Electrodiagnosis of carpal tunnel syndrome in patients with diabetic polyneuropathy. Clinical Neurophysiology 2011;122:1463-1469

6. Landmann G, Stockinger L, Gerber B, Benrath J, Schmelz M, Rukwied R. Local hyperexcitability of C-nociceptors may predict responsiveness to topical lidocaine in neuropathic pain. Plos one 2022;17:e0271327

7. Jonas R, Namer B, Stockinger L, Chisholm K, Schnakenberg M, Landmann G, Kucharczyk M, Konrad C, Schmidt R, Carr R. Tuning in C‐nociceptors to reveal mechanisms in chronic neuropathic pain. Annals of neurology 2018;83:945-957

8. Rolke R, Baron R, Maier Ca, Tölle T, Treede R-D, Beyer A, Binder A, Birbaumer N, Birklein F, Bötefür I. Quantitative sensory testing in the German Research Network on Neuropathic Pain (DFNS): standardized protocol and reference values. Pain® 2006;123:231-243

9. Jende JM, Kender Z, Mooshage C, Groener JB, Alvarez-Ramos L, Kollmer J, Juerchott A, Hahn A, Heiland S, Nawroth P. Diffusion tensor imaging of the sciatic nerve as a surrogate marker for nerve functionality of the upper and lower limb in patients with diabetes and prediabetes. Frontiers in Neuroscience 2021;15:642589

10. Jende JM, Groener JB, Kender Z, Hahn A, Morgenstern J, Heiland S, Nawroth PP, Bendszus M, Kopf S, Kurz FT. Troponin T parallels structural nerve damage in type 2 diabetes: a cross-sectional study using magnetic resonance neurography. Diabetes 2020;69:713-723

11. Ziegler D, Keller J, Maier C, Pannek J. Diabetic neuropathy. Experimental and Clinical Endocrinology & Diabetes 2021;129:S70-S81

12. Zilliox L, Peltier A, Wren P, Anderson A, Smith A, Singleton J, Feldman E, Alexander N, Russell J. Assessing autonomic dysfunction in early diabetic neuropathy: the Survey of Autonomic Symptoms. Neurology 2011;76:1099-1105

13. Borg G. *Borg's perceived exertion and pain scales*. Human kinetics, 1998

14. DeFronzo RA, Tobin JD, Andres R. Glucose clamp technique: a method for quantifying insulin secretion and resistance. American Journal of Physiology-Endocrinology and Metabolism 1979;237:E214

15. Kahl S, Nowotny B, Piepel S, Nowotny PJ, Strassburger K, Herder C, Pacini G, Roden M. Estimates of insulin sensitivity from the intravenous-glucose-modified-clamp test depend on suppression of lipolysis in type 2 diabetes: a randomised controlled trial. Diabetologia 2014;57:2094-2102

16. Bischof MG, Bernroider E, Krssak M, Krebs M, Stingl H, Nowotny P, Yu C, Shulman GI, Waldhäusl W, Roden M. Hepatic glycogen metabolism in type 1 diabetes after long-term near normoglycemia. Diabetes 2002;51:49-54

17. Kahl S, Strassburger K, Nowotny B, Livingstone R, Klüppelholz B, Kessel K, Hwang J-H, Giani G, Hoffmann B, Pacini G. Comparison of liver fat indices for the diagnosis of hepatic steatosis and insulin resistance. PloS one 2014;9:e94059

18. Storlien L, Oakes ND, Kelley DE. Metabolic flexibility. Proceedings of the Nutrition Society 2004;63:363-368

19. Schadewaldt P, Nowotny B, Straßburger K, Kotzka J, Roden M. Indirect calorimetry in humans: a postcalorimetric evaluation procedure for correction of metabolic monitor variability. The American journal of clinical nutrition 2013;97:763-773

20. Ziegler D, Papanas N, Zhivov A, Allgeier S, Winter K, Ziegler I, Brueggemann J, Strom A, Peschel S, Koehler B. Early detection of nerve fiber loss by corneal confocal microscopy and skin biopsy in recently diagnosed type 2 diabetes. Diabetes 2014;63:2454-2463

21. Ware JE, Kosinski M, Keller SD. A 12-Item Short-Form Health Survey: construction of scales and preliminary tests of reliability and validity. Medical care 1996;34:220-233

22. Gräfe K, Zipfel S, Herzog W, Löwe B. Screening psychischer Störungen mit dem “Gesundheitsfragebogen für Patienten (PHQ-D)“. Diagnostica 2004;50:171-181
